# Supplementary figures and images for: Replication stress-inducing ELF3 upregulation promotes BRCA1-deficient breast tumorigenesis in luminal progenitors
Source: eLife. 2026 Jan 7;12:RP89573. doi: 10.7554/eLife.89573 (PMC12779267; doi:10.7554/eLife.89573)

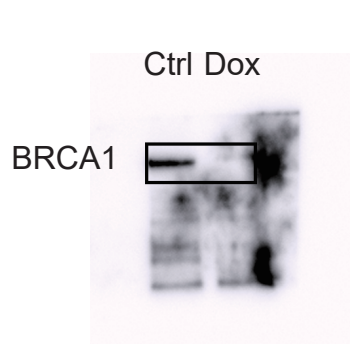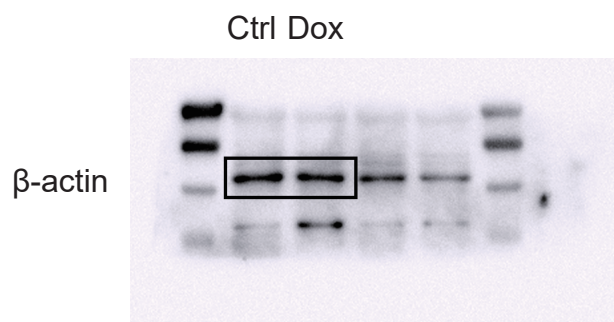

Supplement: Figure 1—figure supplement 1—source data 1. [file elife-89573-fig1-figsupp1-data1.zip › Figure 1 Supplement 1C.pdf]

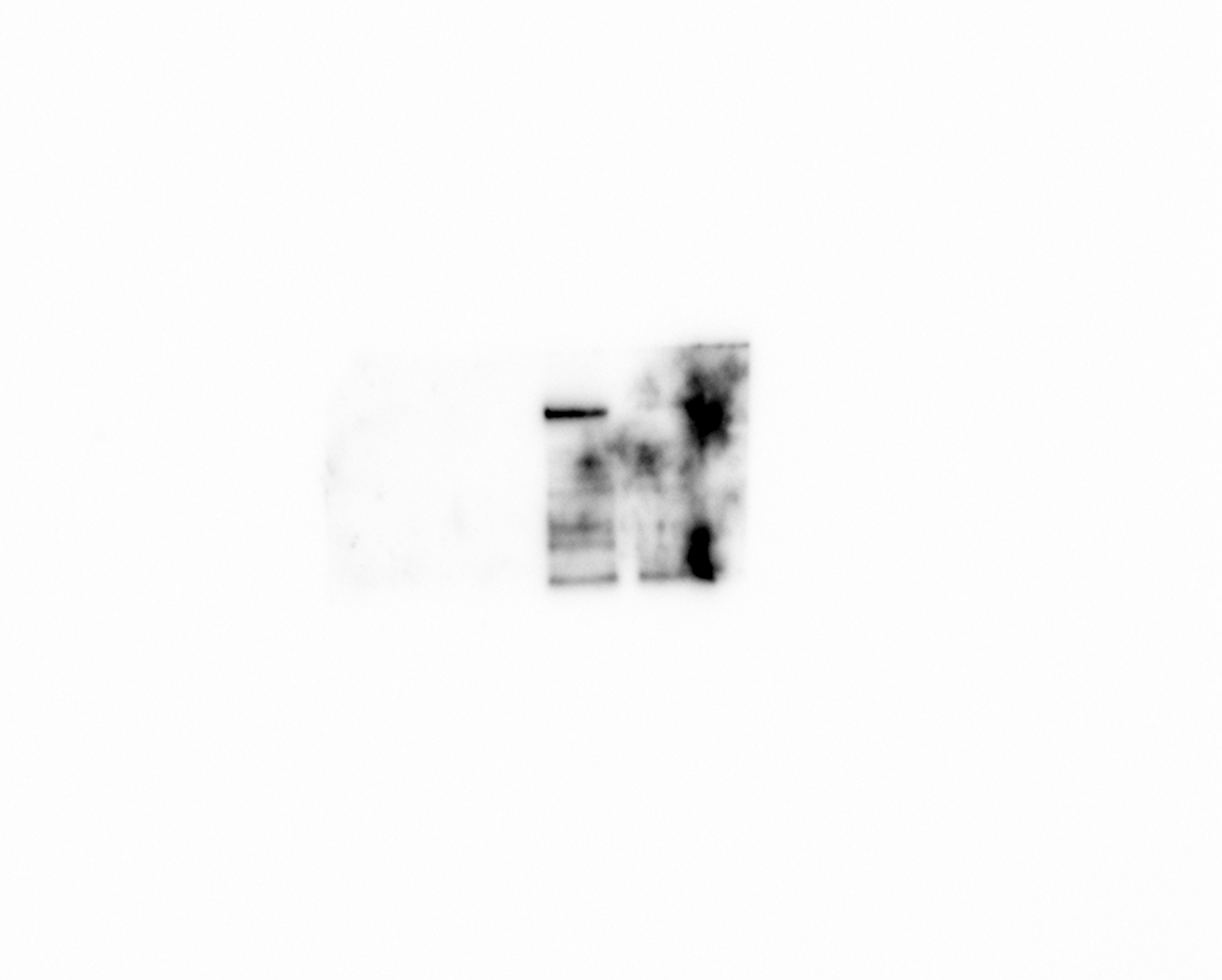

Supplement: Figure 1—figure supplement 1—source data 2. [file elife-89573-fig1-figsupp1-data2.zip › Figure 1 Supplement 1C/BRCA1.Tif]

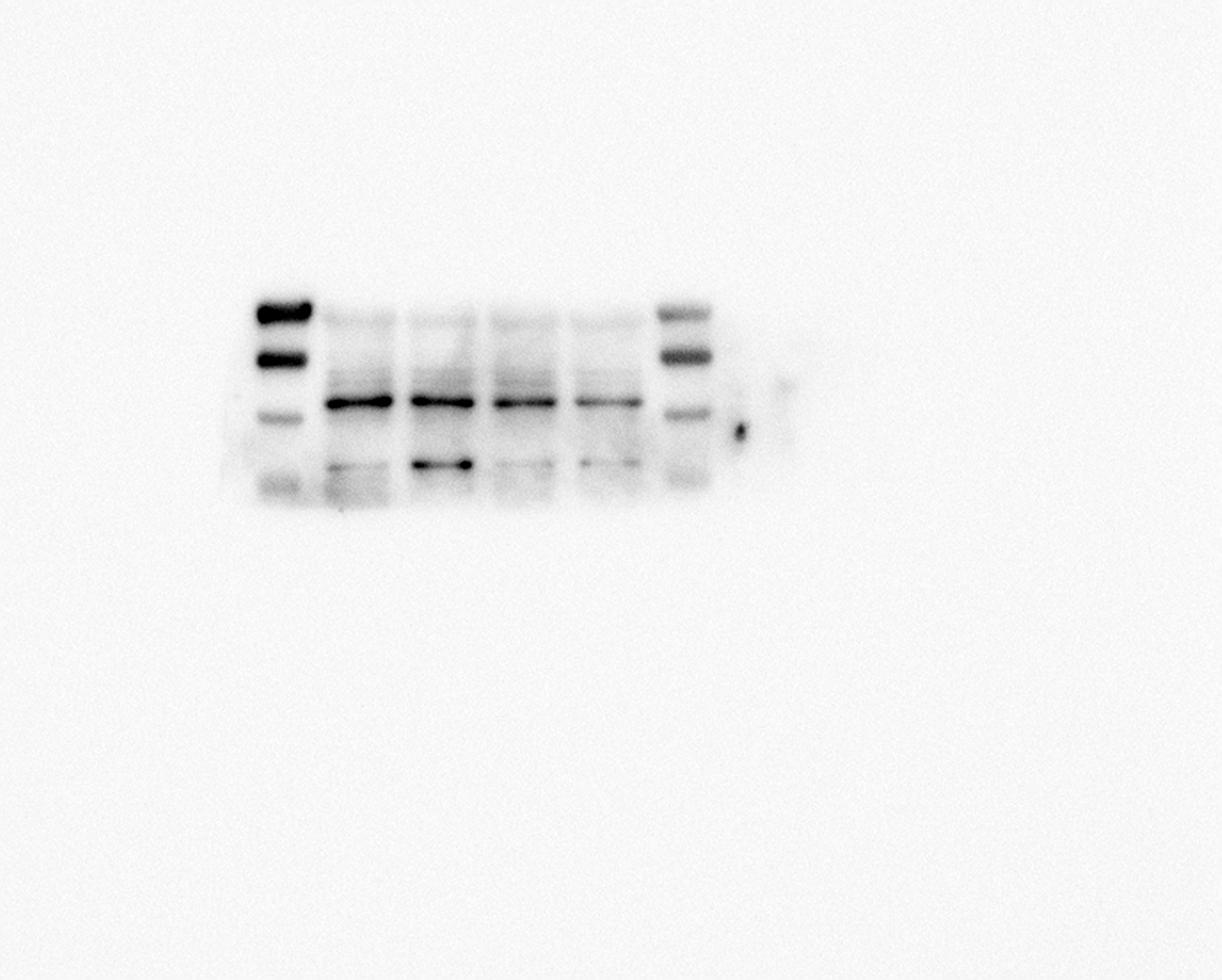

Supplement: Figure 1—figure supplement 1—source data 2. [file elife-89573-fig1-figsupp1-data2.zip › Figure 1 Supplement 1C/β-actin.Tif]

Figure 3C

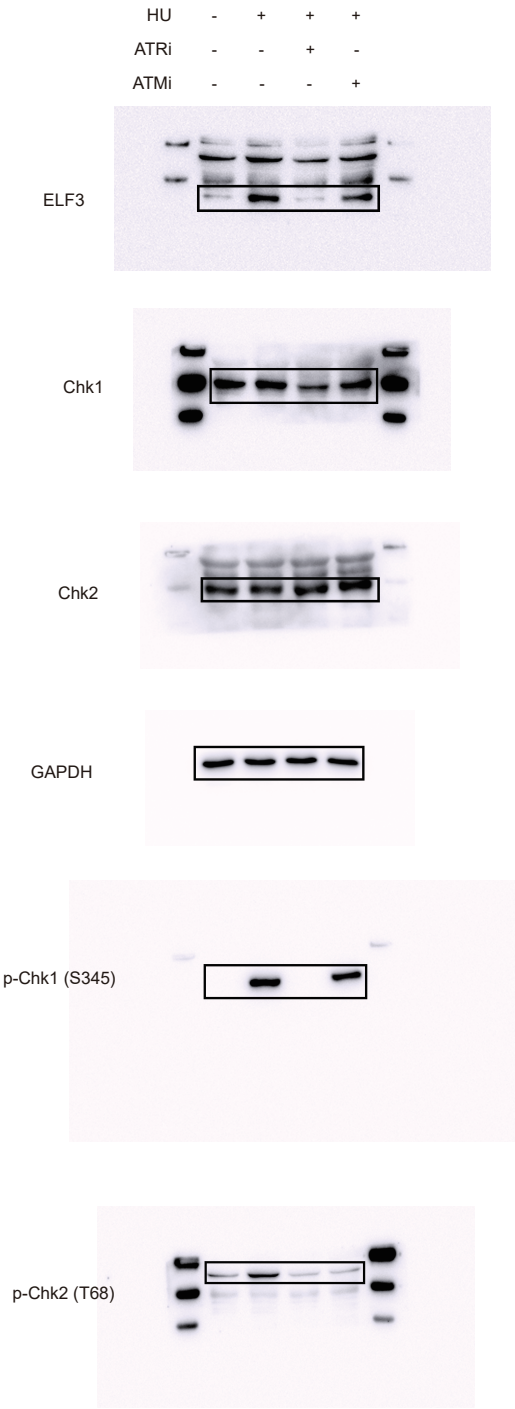

Figure 3D

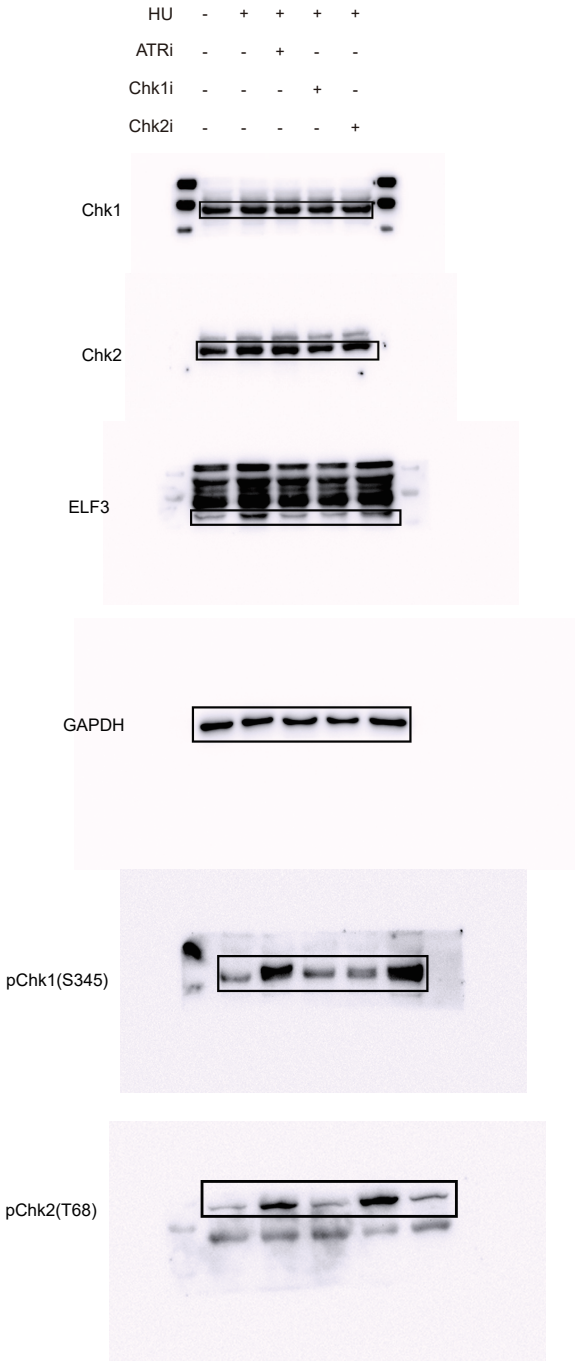

Figure 3E

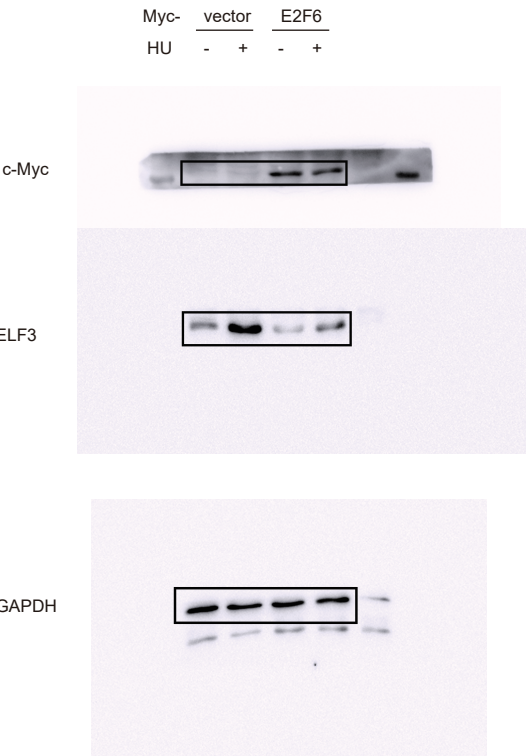

Figure 3L

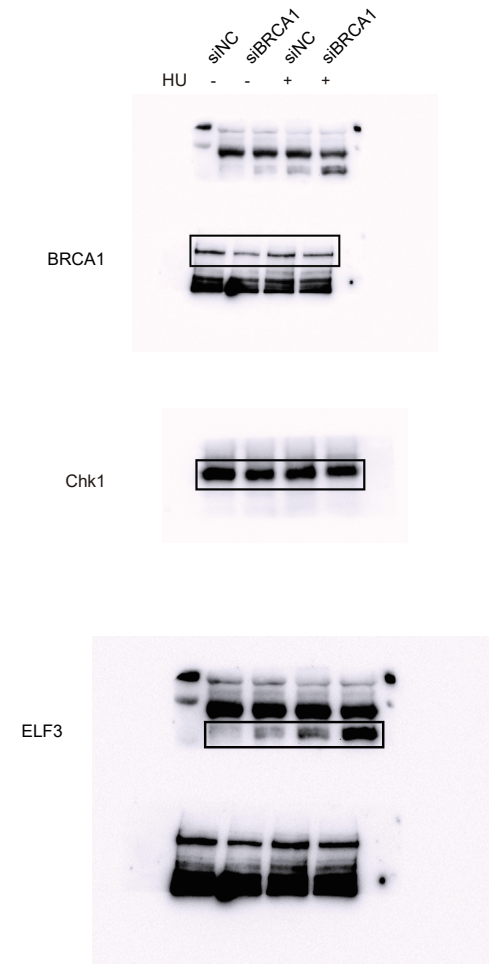

Figure 3H

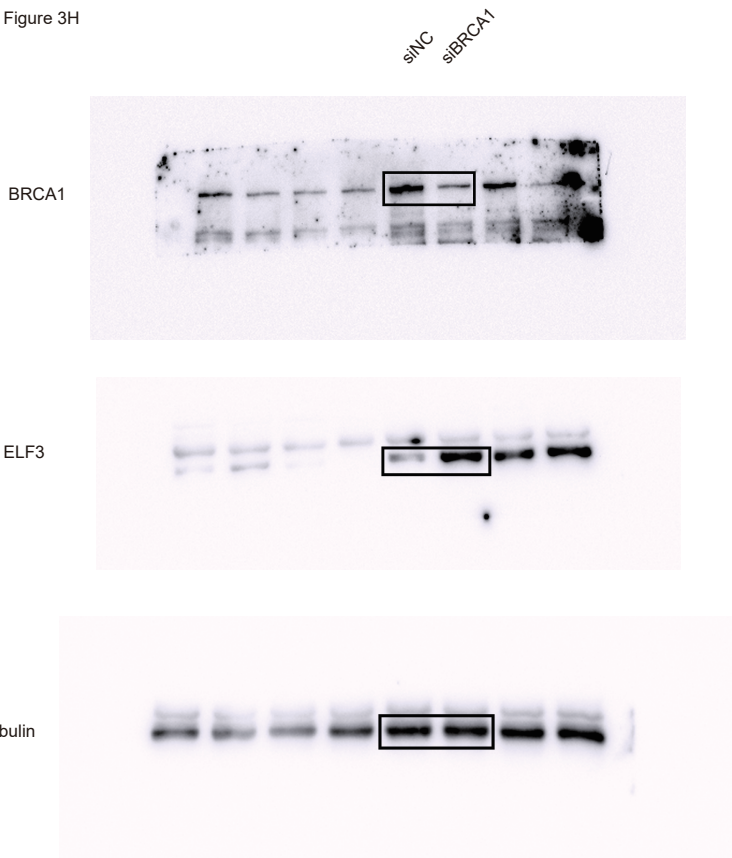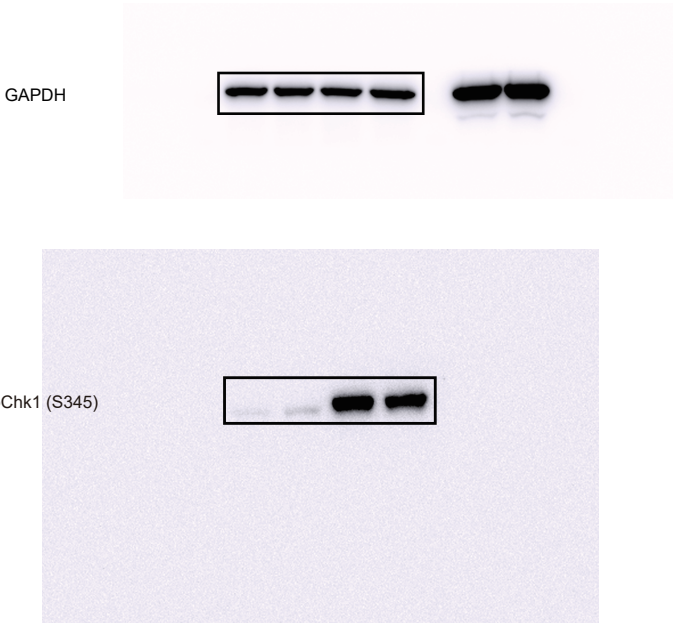

Supplement: Figure 3—source data 1. [file elife-89573-fig3-data1.zip › Figure 3-Source data.pdf]

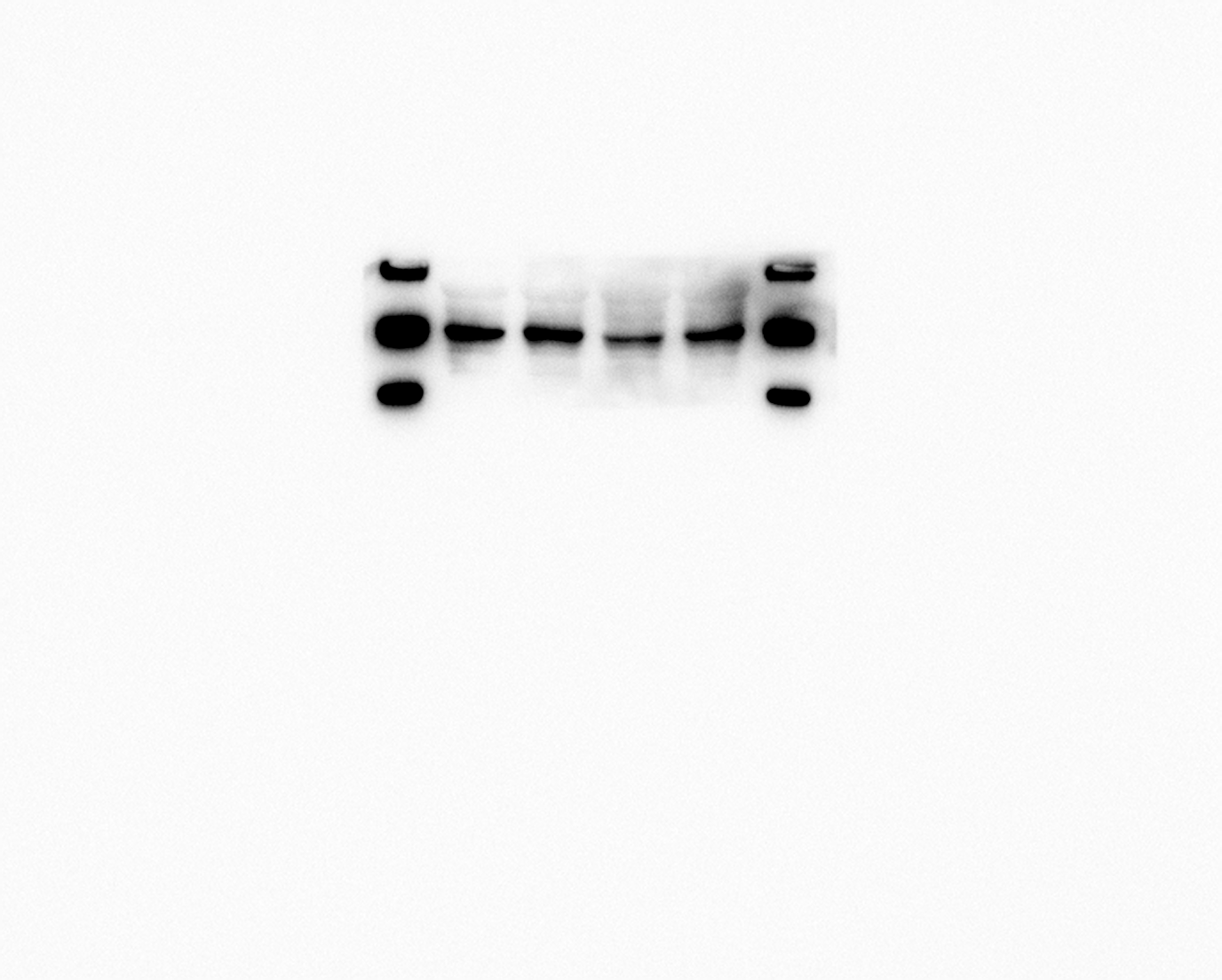

Supplement: Figure 3—source data 2. [file elife-89573-fig3-data2.zip › Figure 3C/Chk1.Tif]

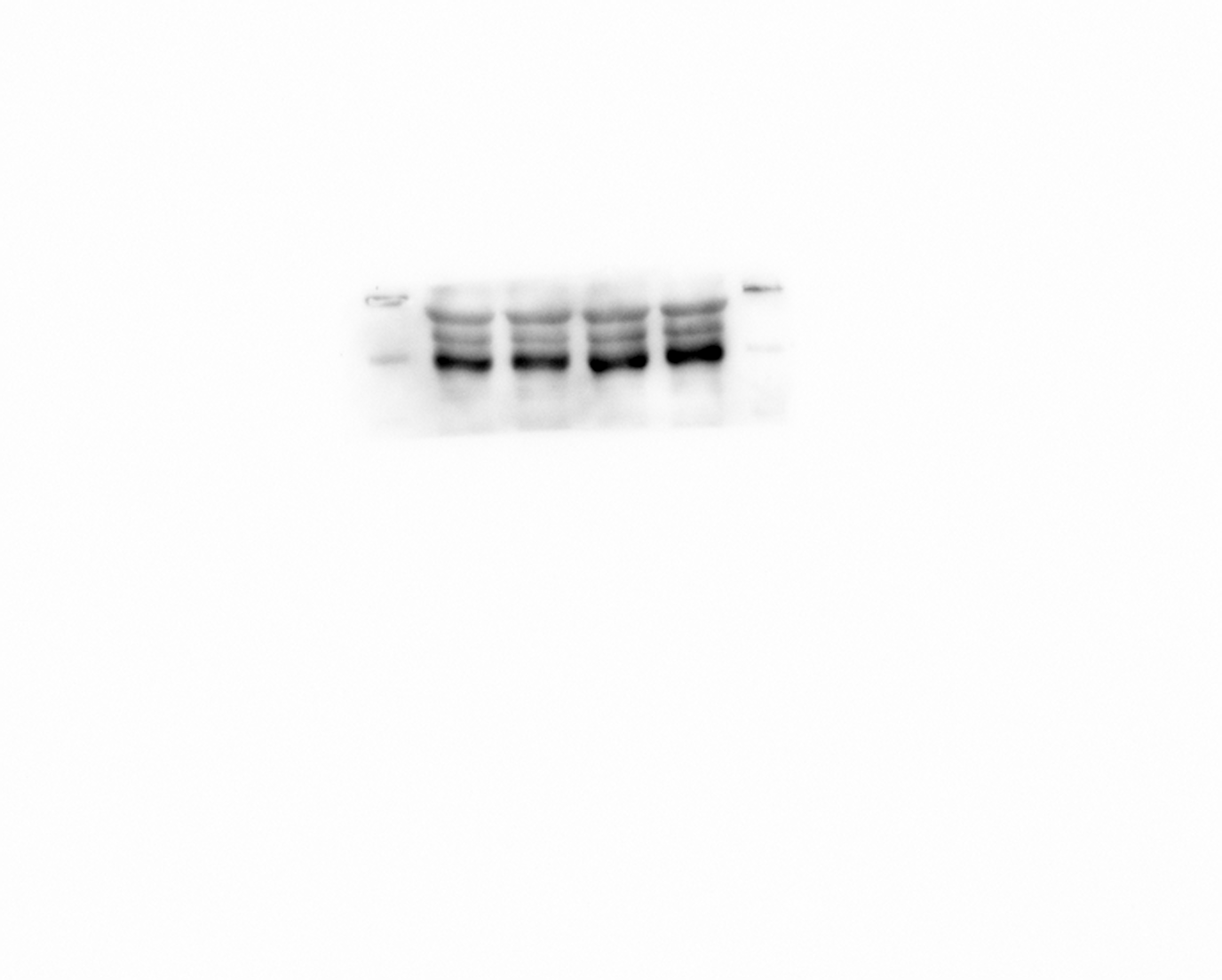

Supplement: Figure 3—source data 2. [file elife-89573-fig3-data2.zip › Figure 3C/Chk2.Tif]

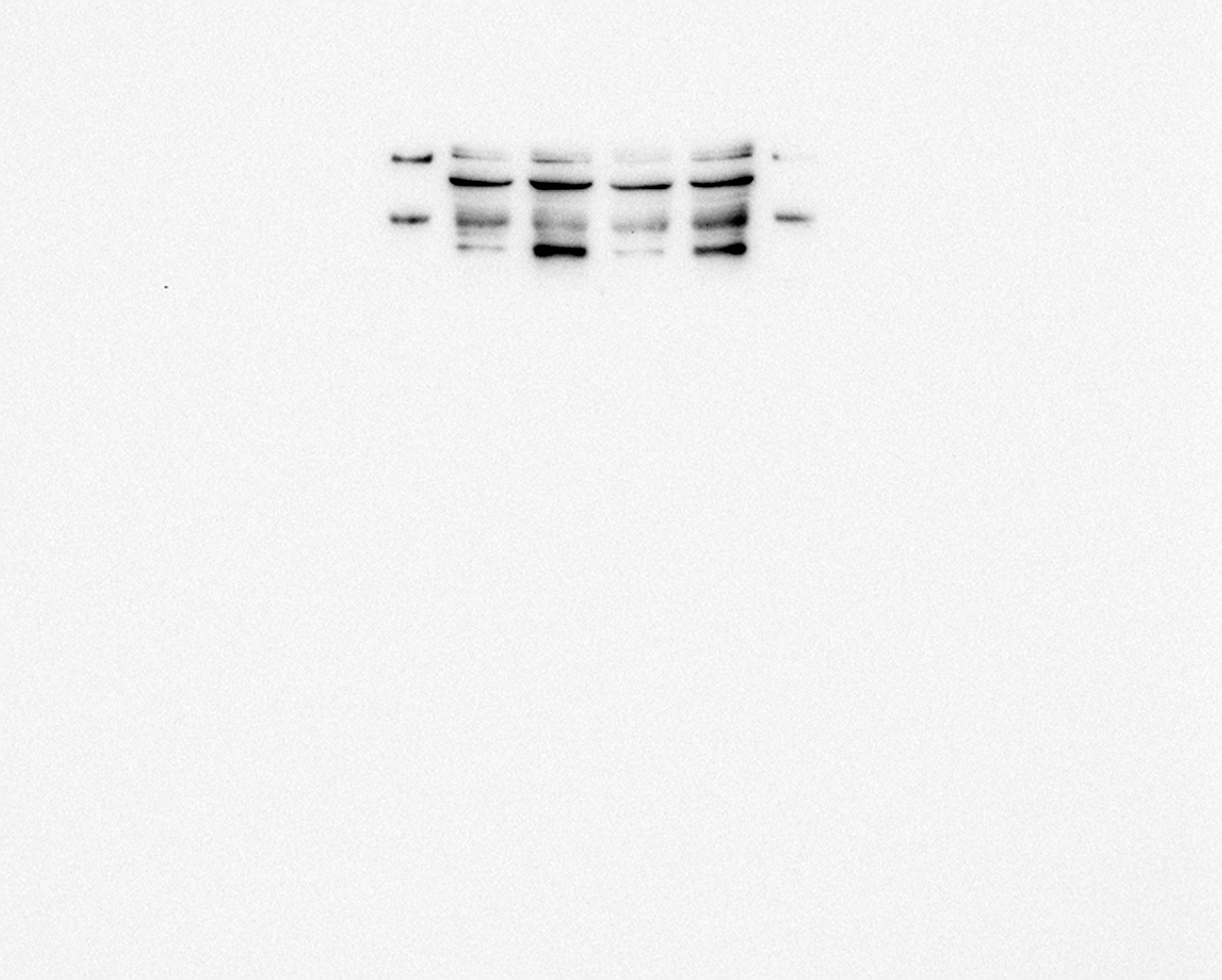

Supplement: Figure 3—source data 2. [file elife-89573-fig3-data2.zip › Figure 3C/ELF3.Tif]

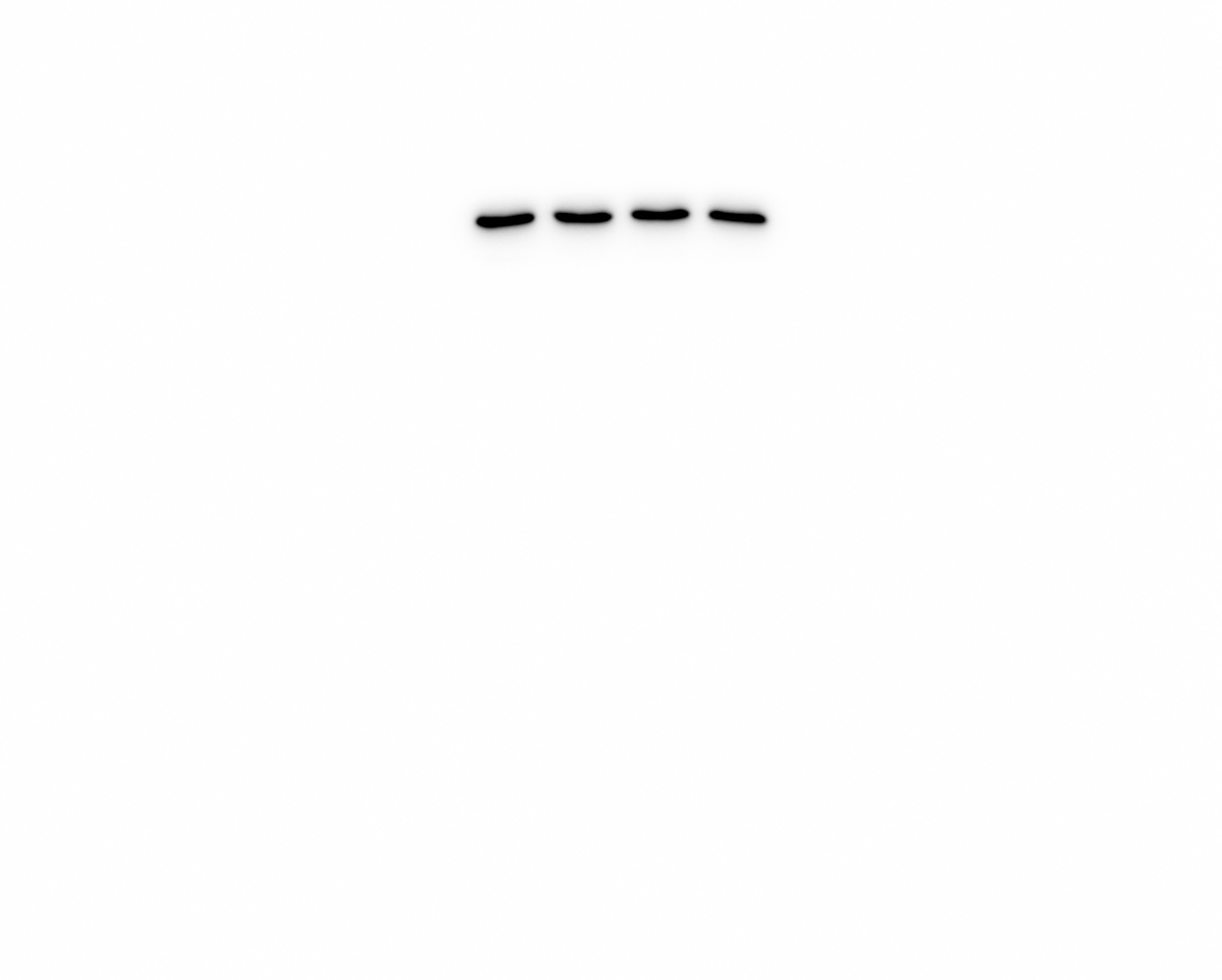

Supplement: Figure 3—source data 2. [file elife-89573-fig3-data2.zip › Figure 3C/GAPDH.Tif]

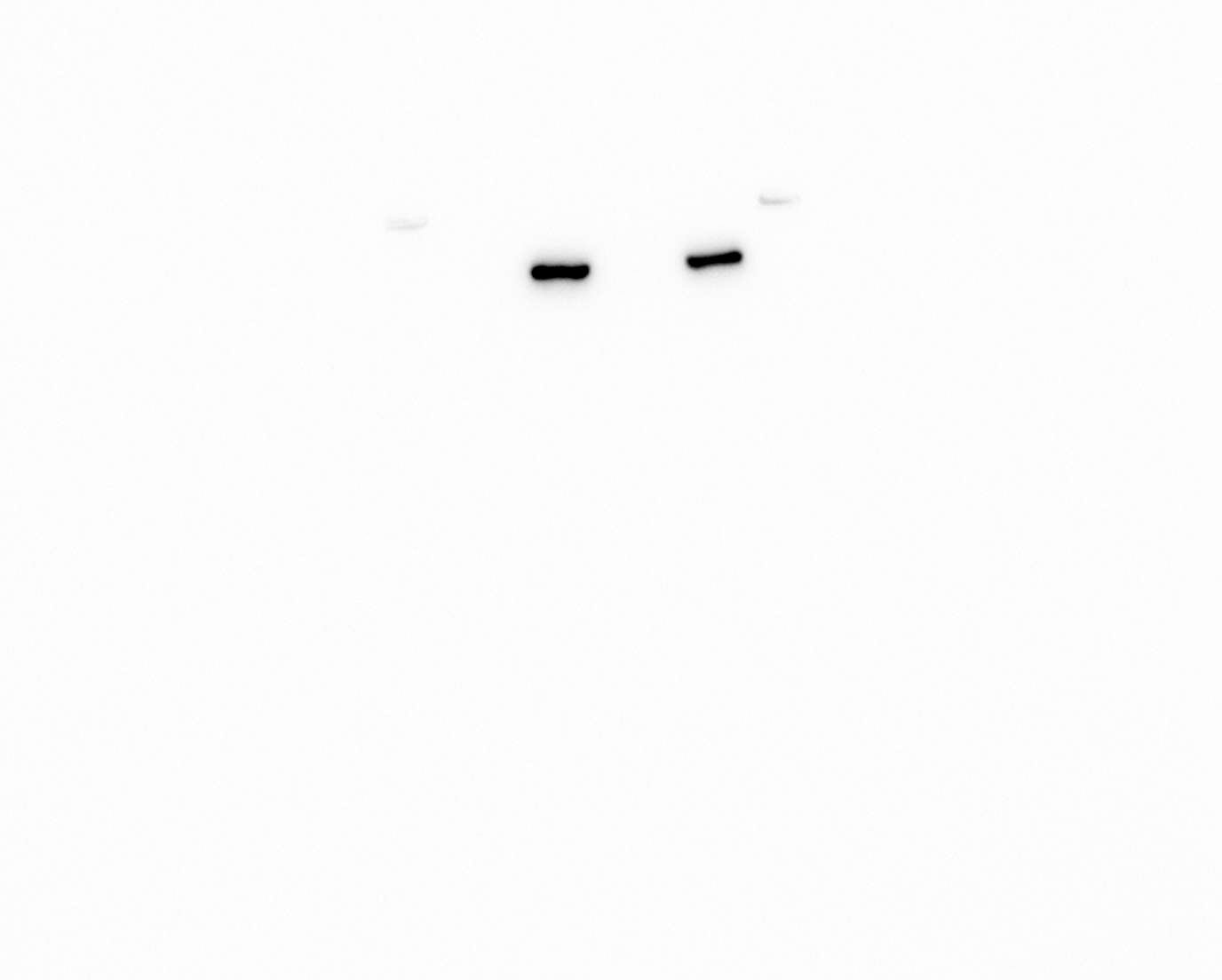

Supplement: Figure 3—source data 2. [file elife-89573-fig3-data2.zip › Figure 3C/p-Chk1.Tif]

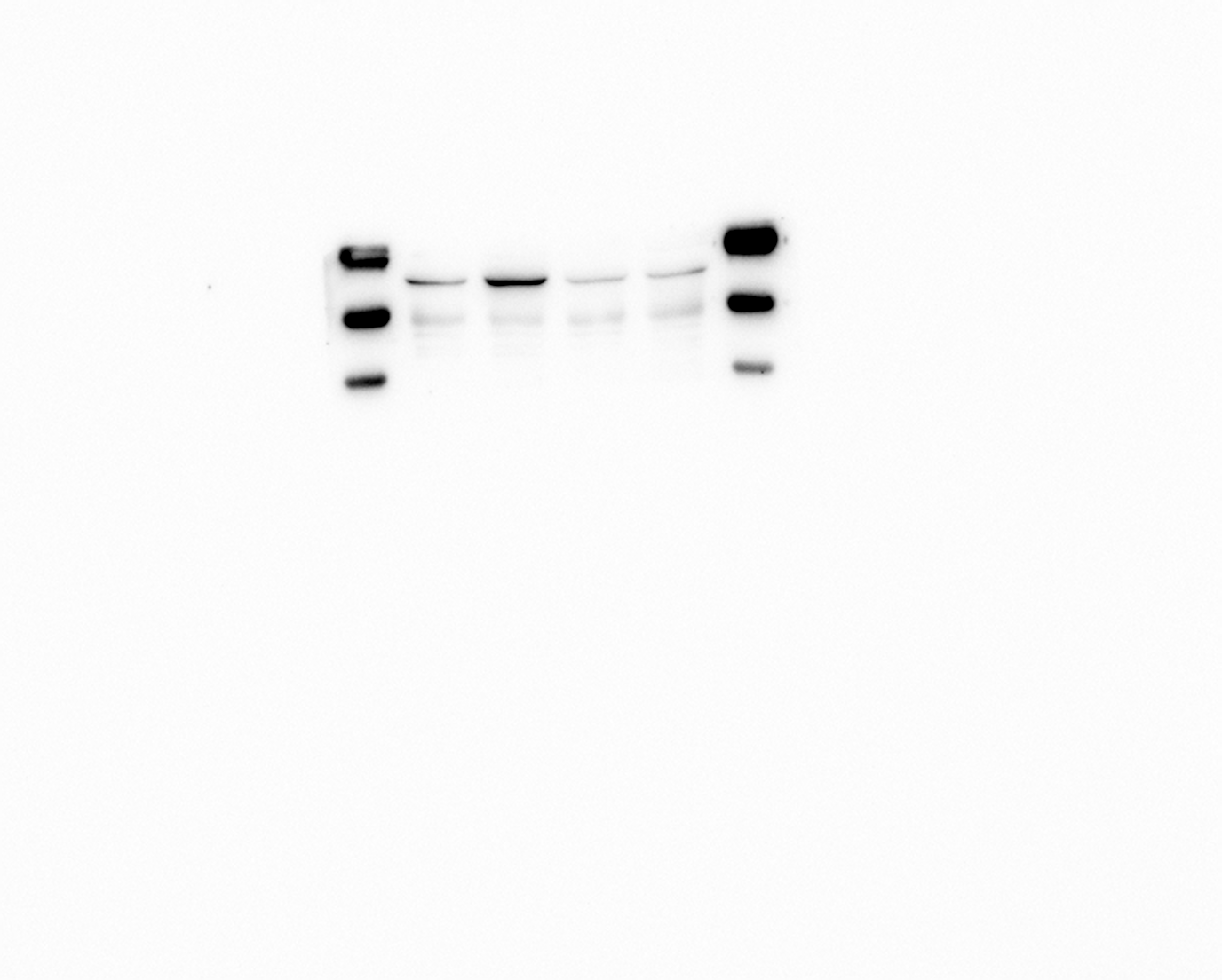

Supplement: Figure 3—source data 2. [file elife-89573-fig3-data2.zip › Figure 3C/p-Chk2.Tif]

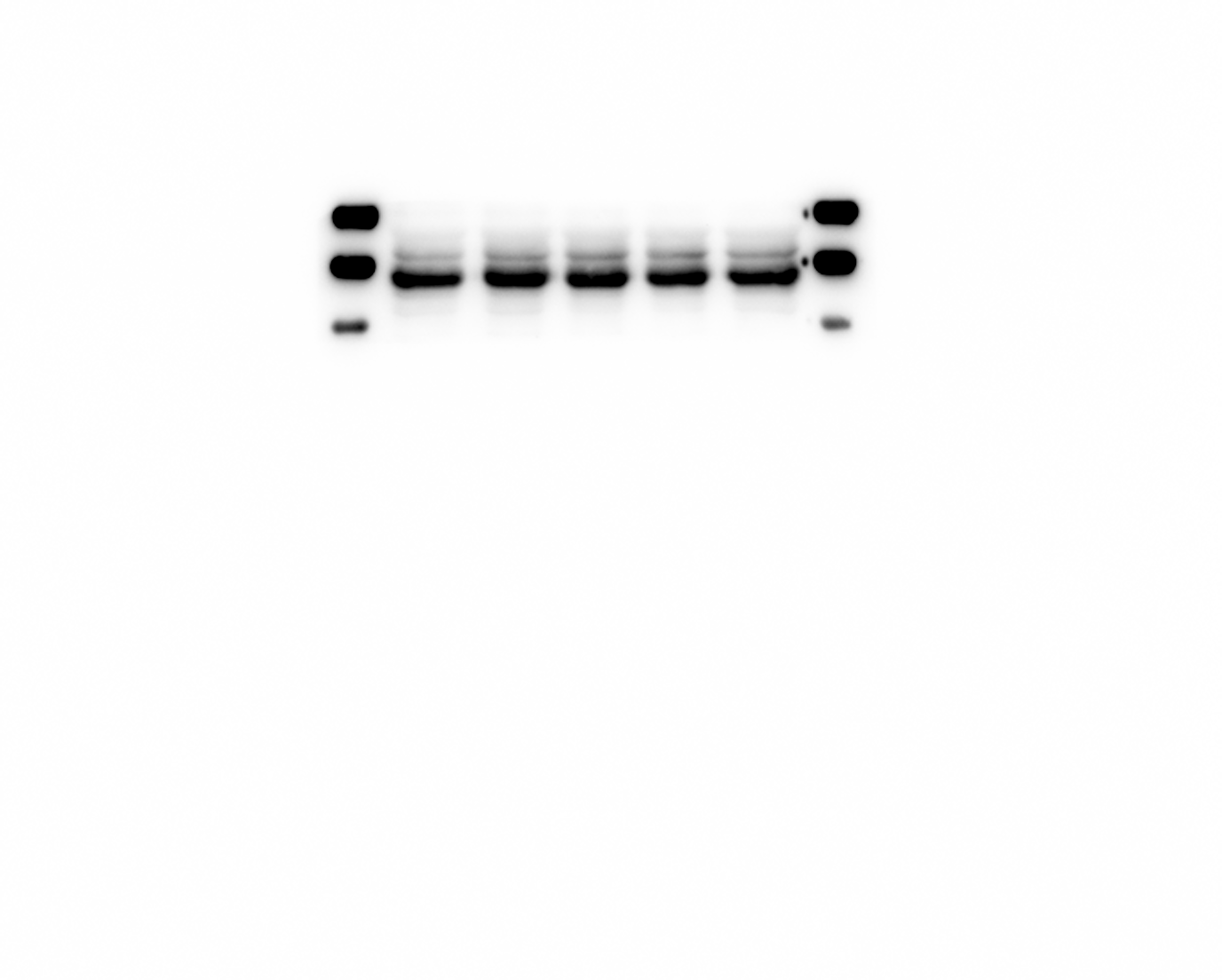

Supplement: Figure 3—source data 2. [file elife-89573-fig3-data2.zip › Figure 3D/Chk1.Tif]

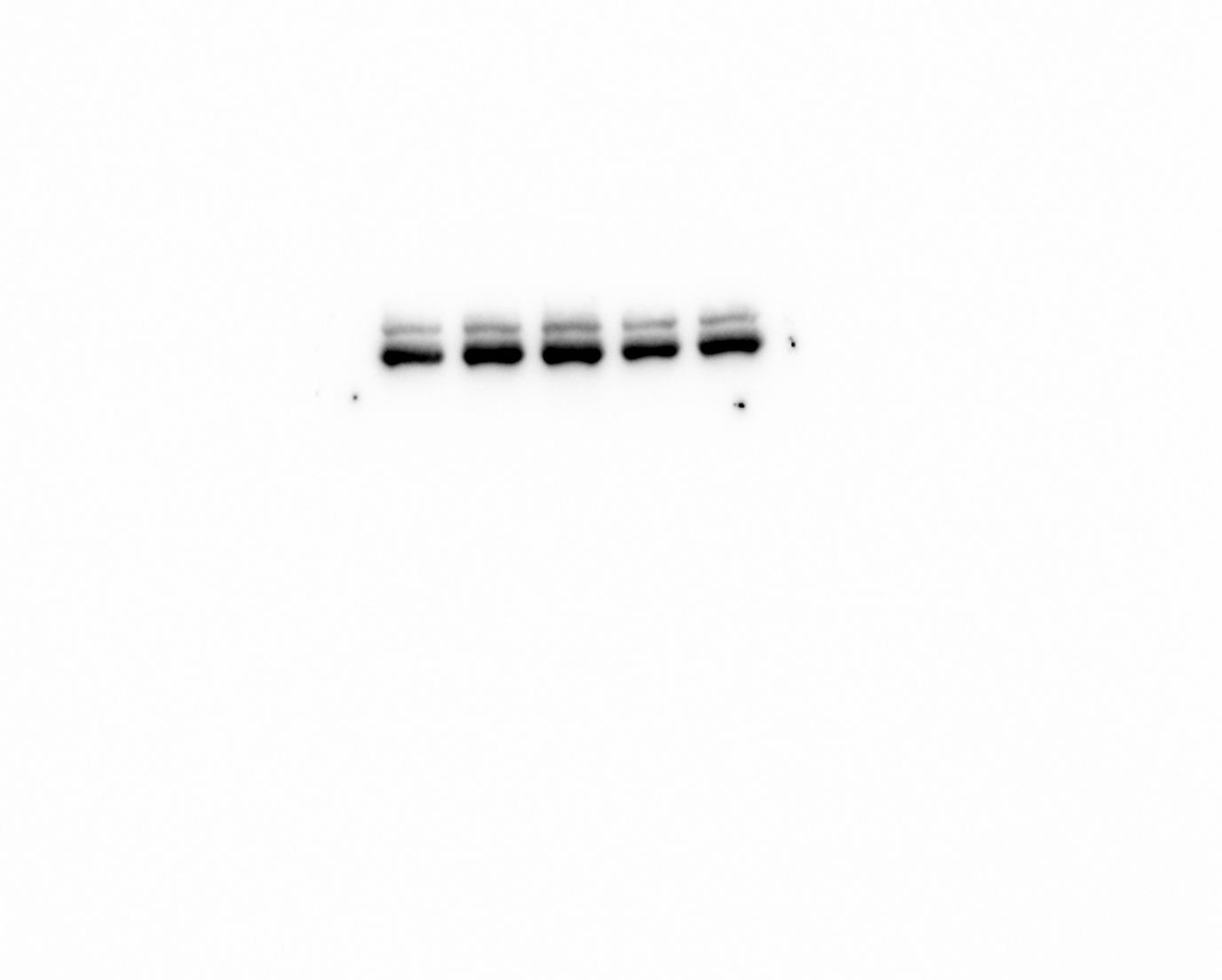

Supplement: Figure 3—source data 2. [file elife-89573-fig3-data2.zip › Figure 3D/Chk2.Tif]

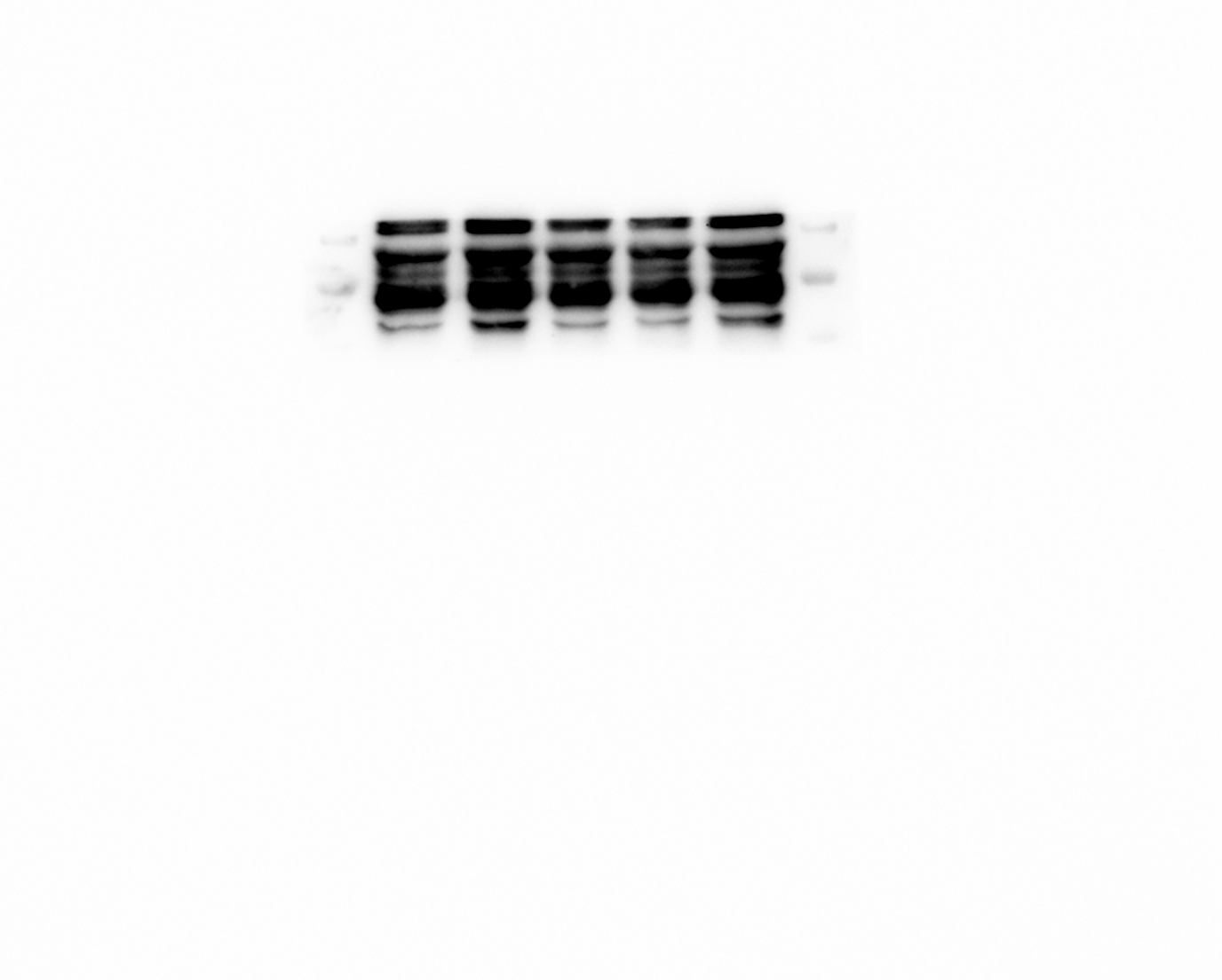

Supplement: Figure 3—source data 2. [file elife-89573-fig3-data2.zip › Figure 3D/ELF3.Tif]

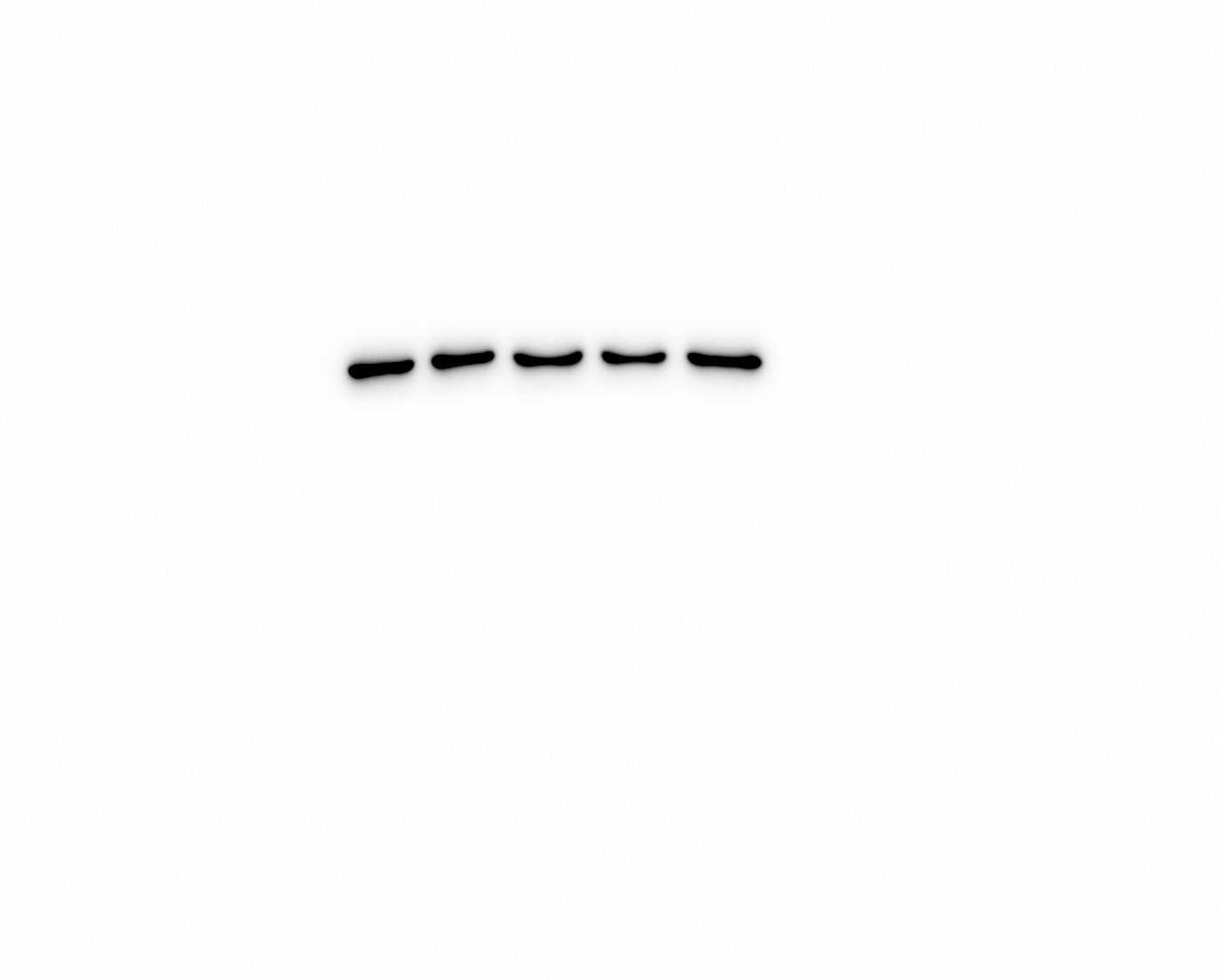

Supplement: Figure 3—source data 2. [file elife-89573-fig3-data2.zip › Figure 3D/GAPDH.Tif]

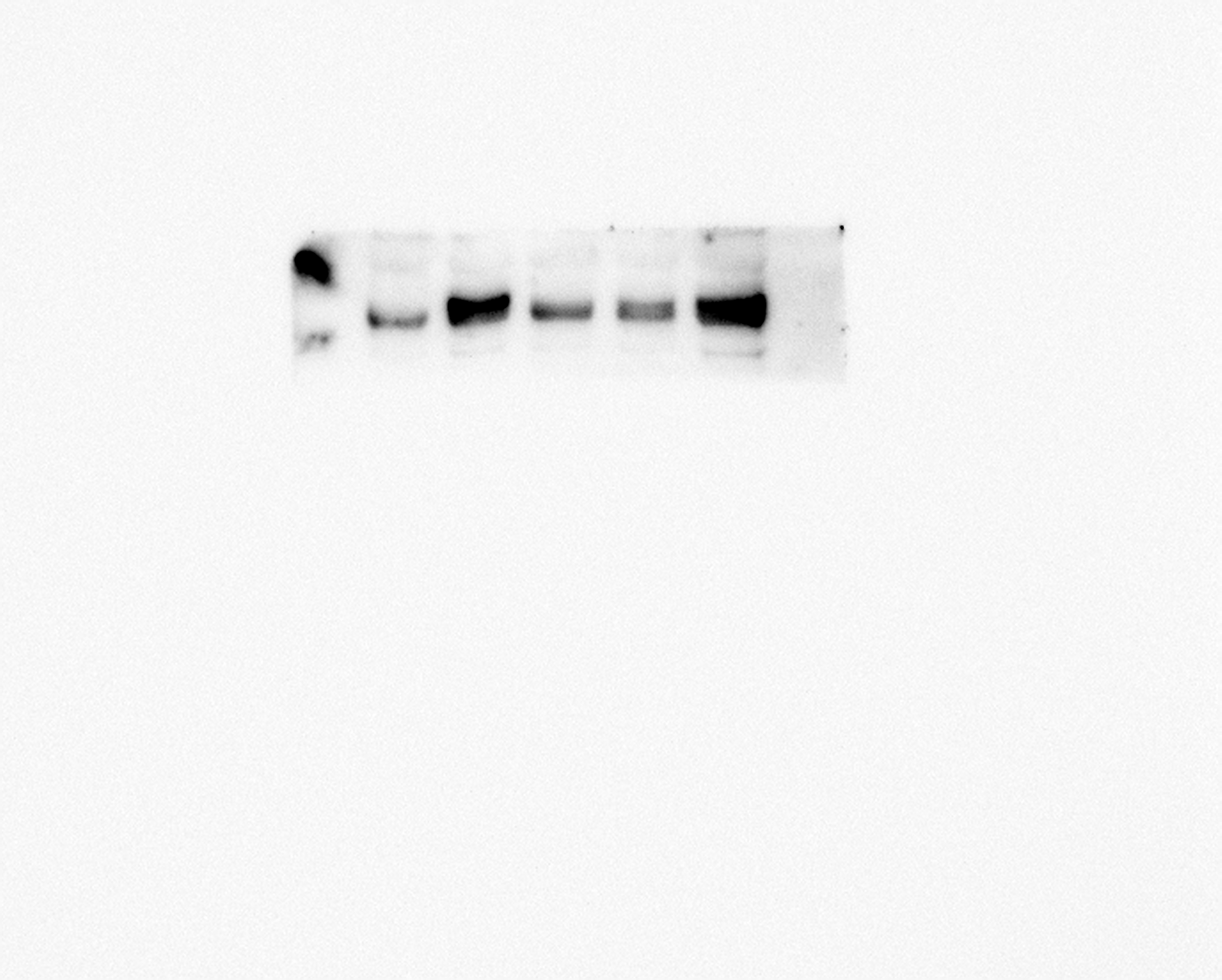

Supplement: Figure 3—source data 2. [file elife-89573-fig3-data2.zip › Figure 3D/p-Chk1.Tif]

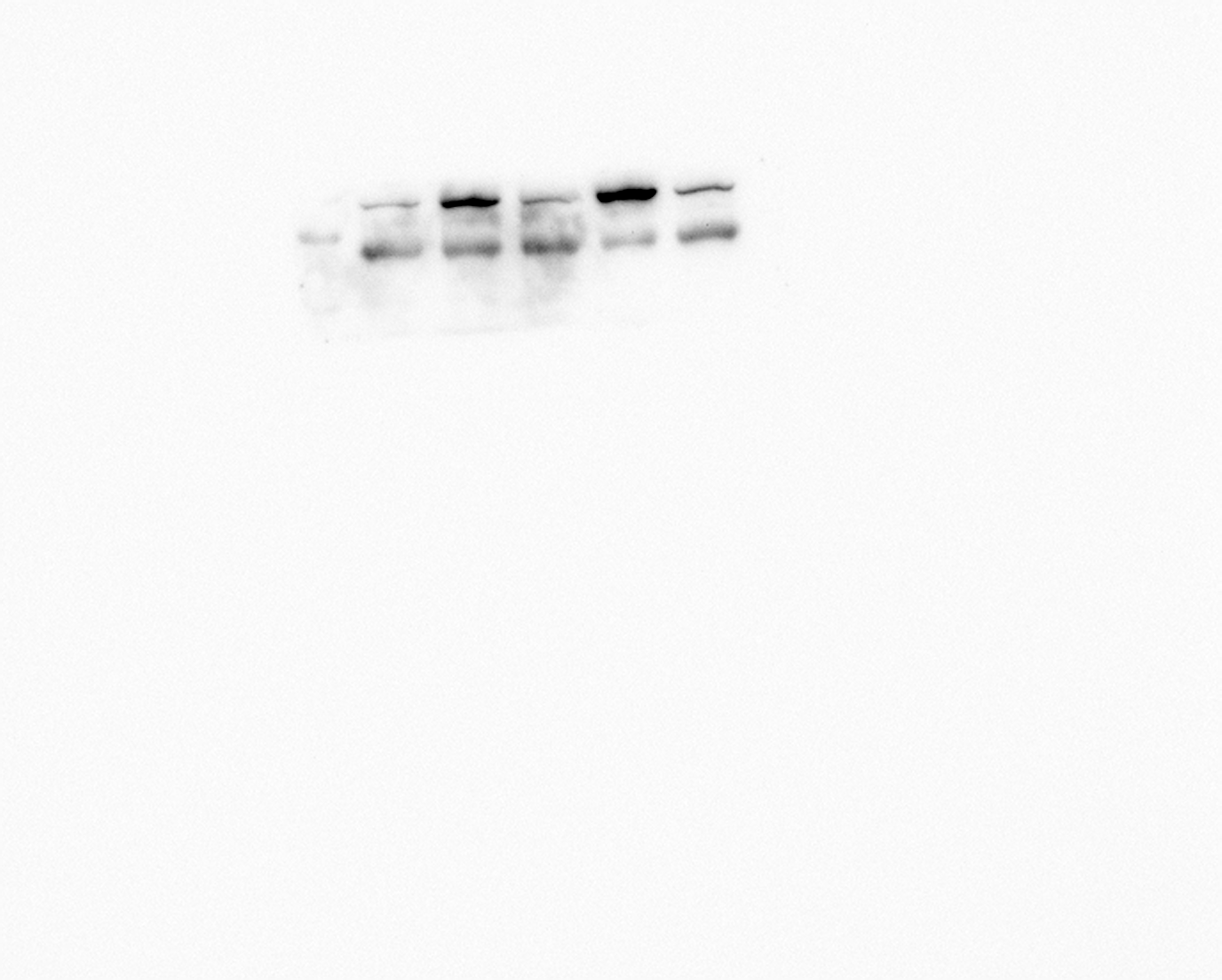

Supplement: Figure 3—source data 2. [file elife-89573-fig3-data2.zip › Figure 3D/p-Chk2.Tif]

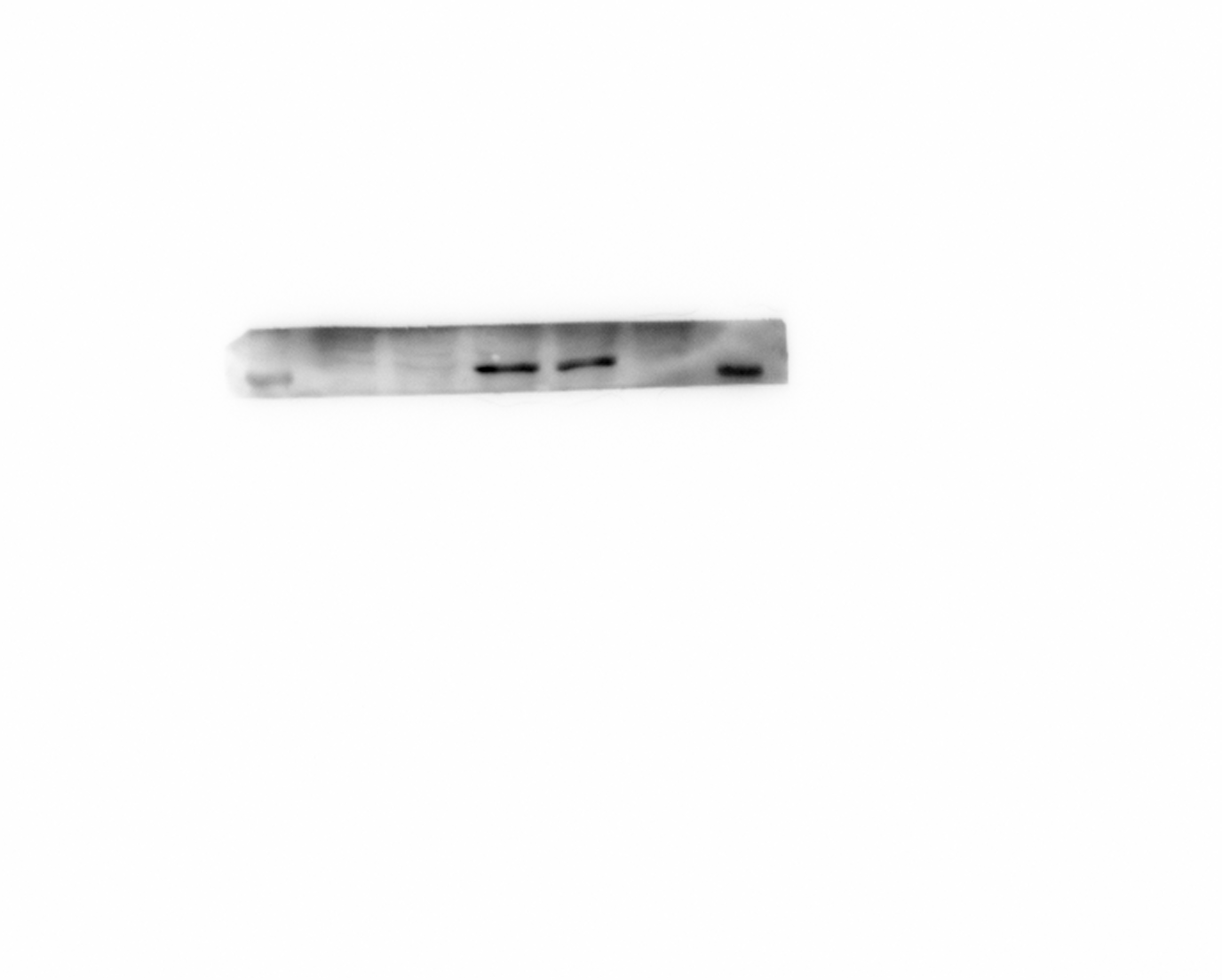

Supplement: Figure 3—source data 2. [file elife-89573-fig3-data2.zip › Figure 3E/c-Myc.Tif]

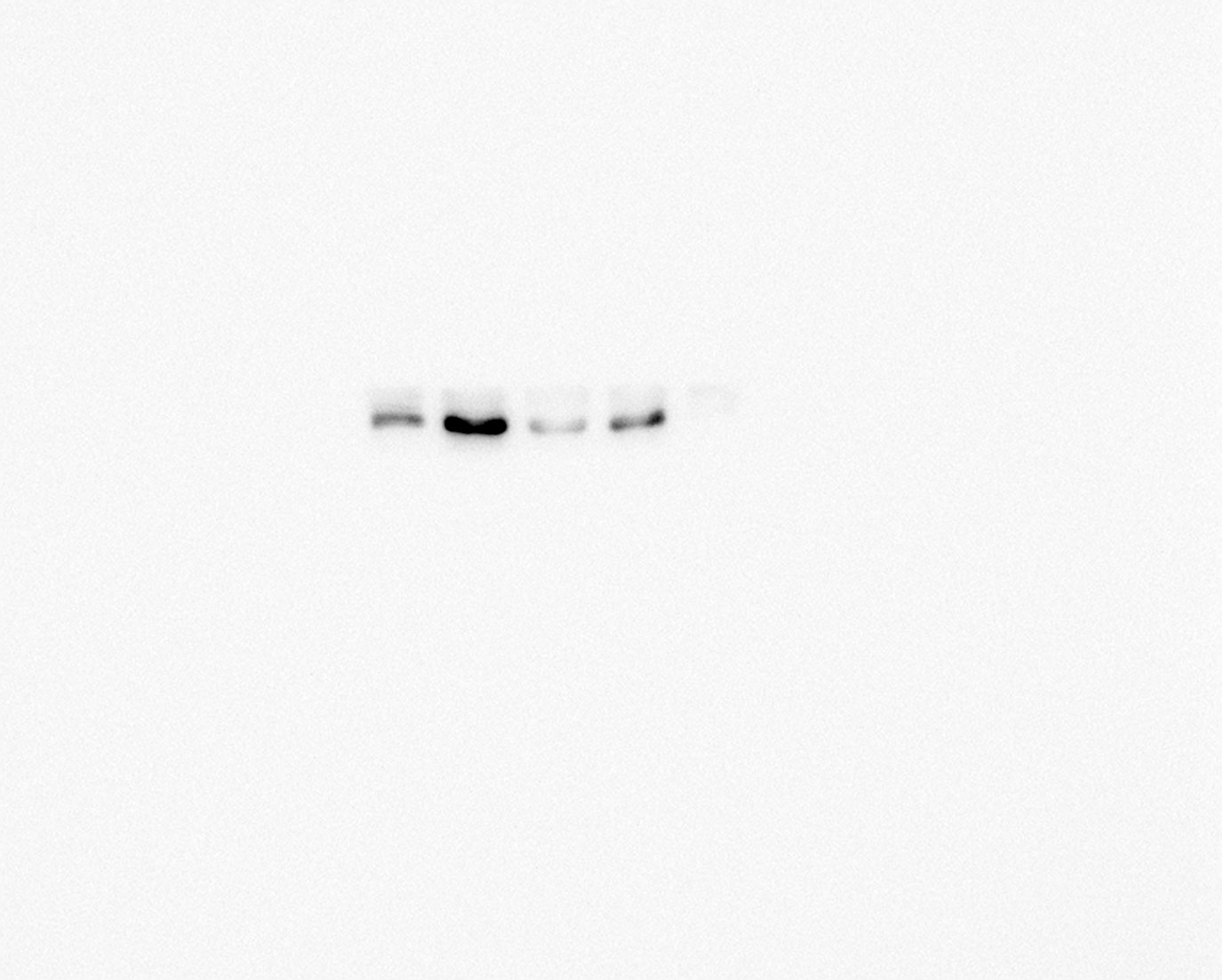

Supplement: Figure 3—source data 2. [file elife-89573-fig3-data2.zip › Figure 3E/ELF3.Tif]

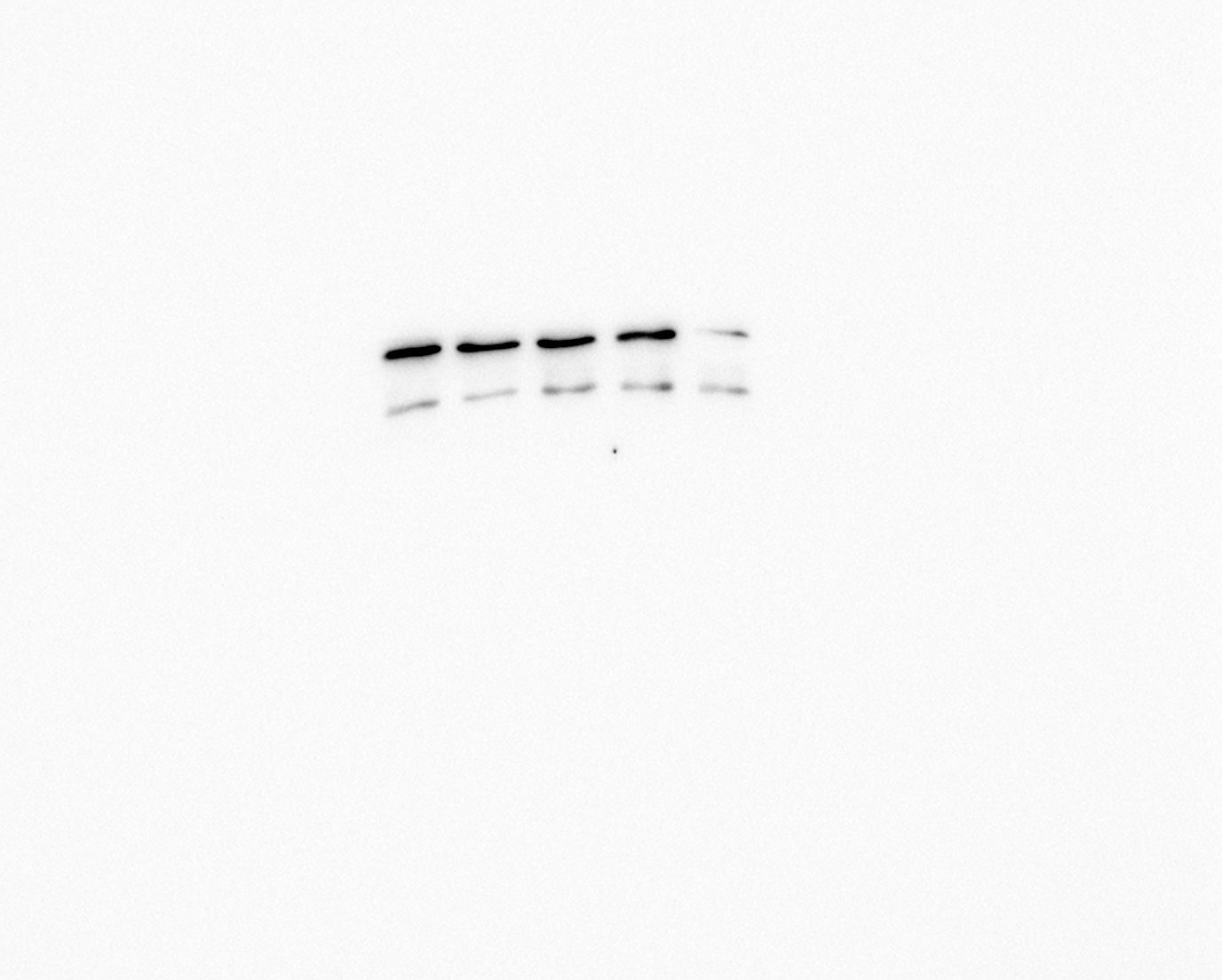

Supplement: Figure 3—source data 2. [file elife-89573-fig3-data2.zip › Figure 3E/GAPDH.Tif]

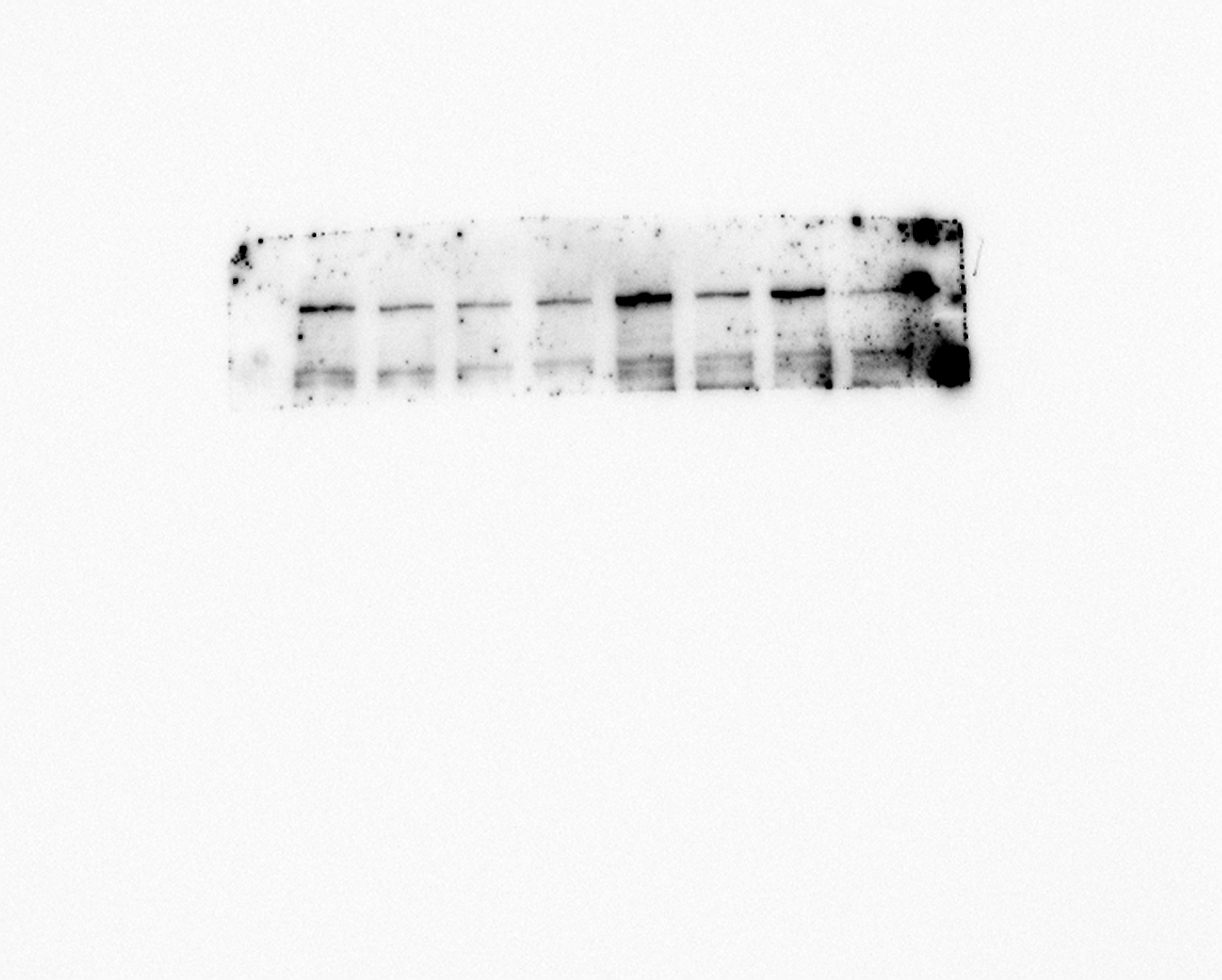

Supplement: Figure 3—source data 2. [file elife-89573-fig3-data2.zip › Figure 3H/BRCA1.Tif]

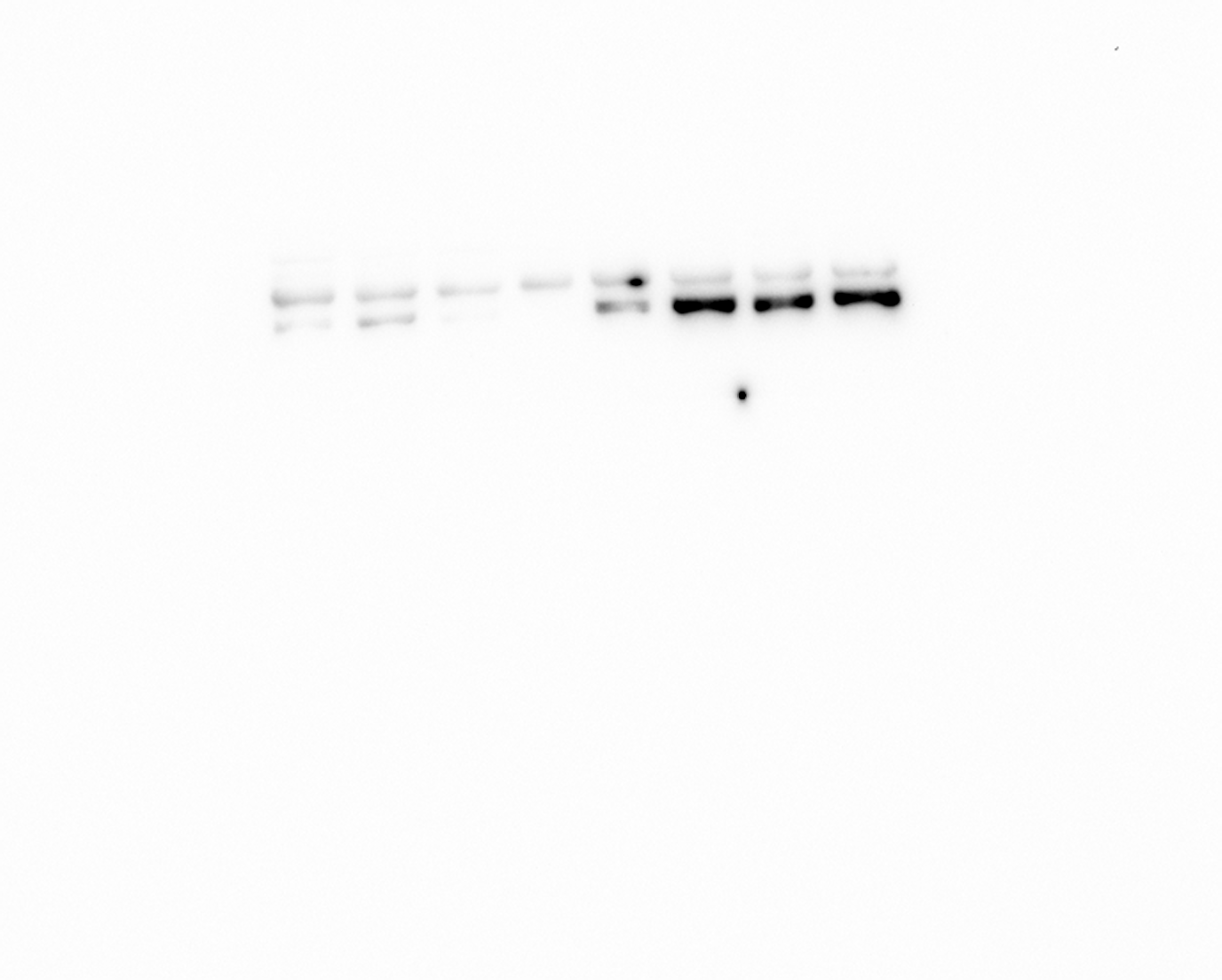

Supplement: Figure 3—source data 2. [file elife-89573-fig3-data2.zip › Figure 3H/ELF3.Tif]

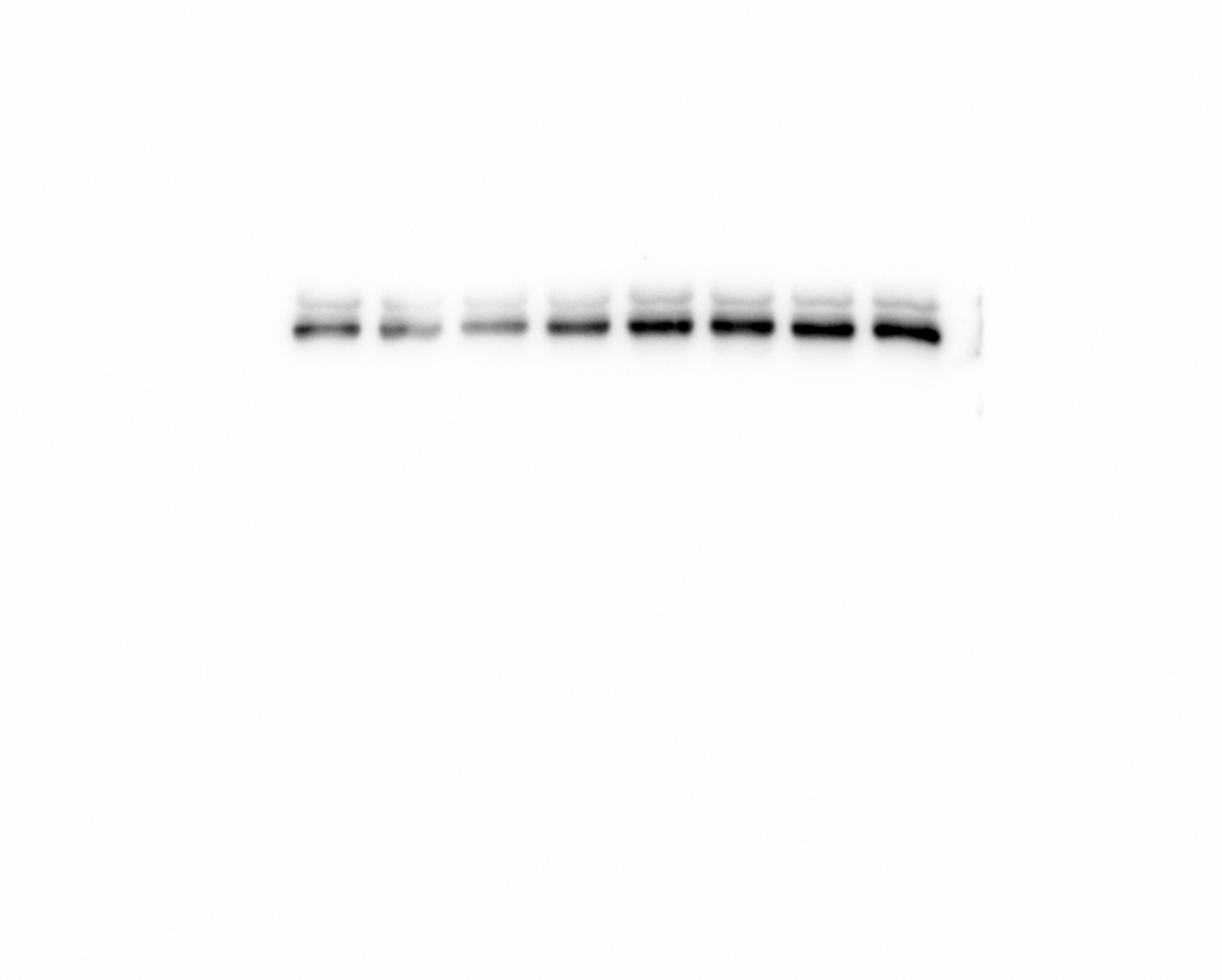

Supplement: Figure 3—source data 2. [file elife-89573-fig3-data2.zip › Figure 3H/β-tubulin.Tif]

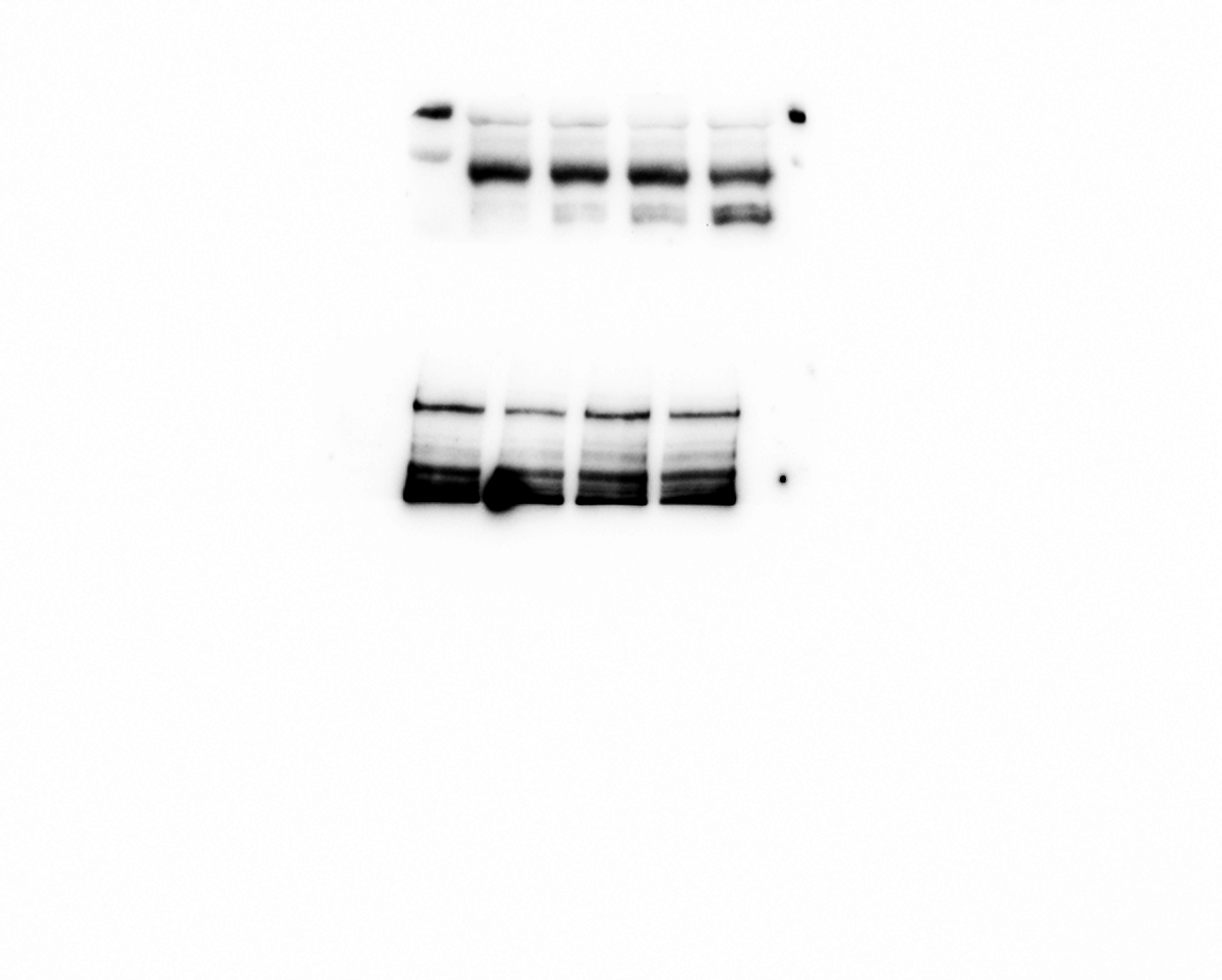

Supplement: Figure 3—source data 2. [file elife-89573-fig3-data2.zip › Figure 3L/BRCA1.tif]

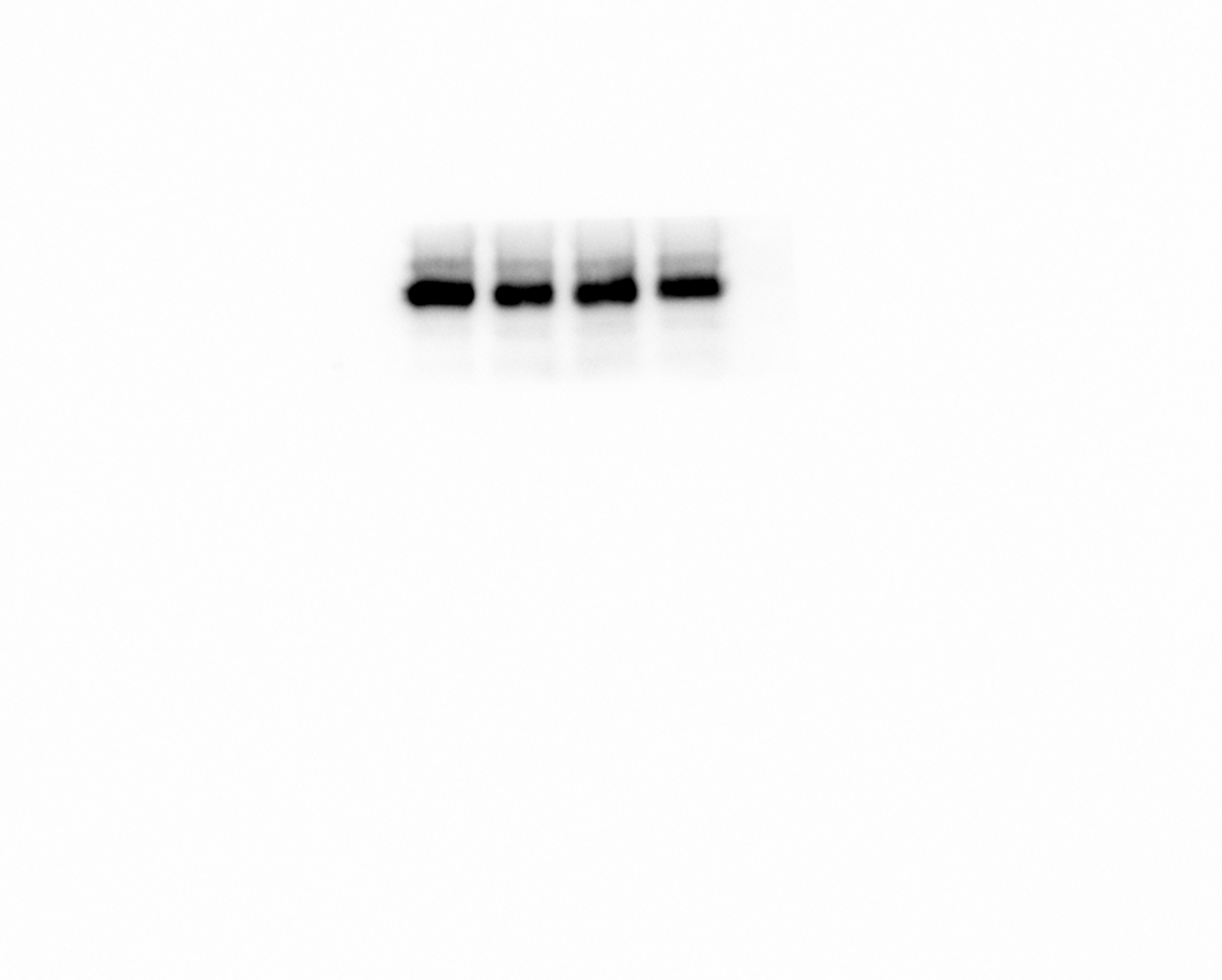

Supplement: Figure 3—source data 2. [file elife-89573-fig3-data2.zip › Figure 3L/Chk1.Tif]

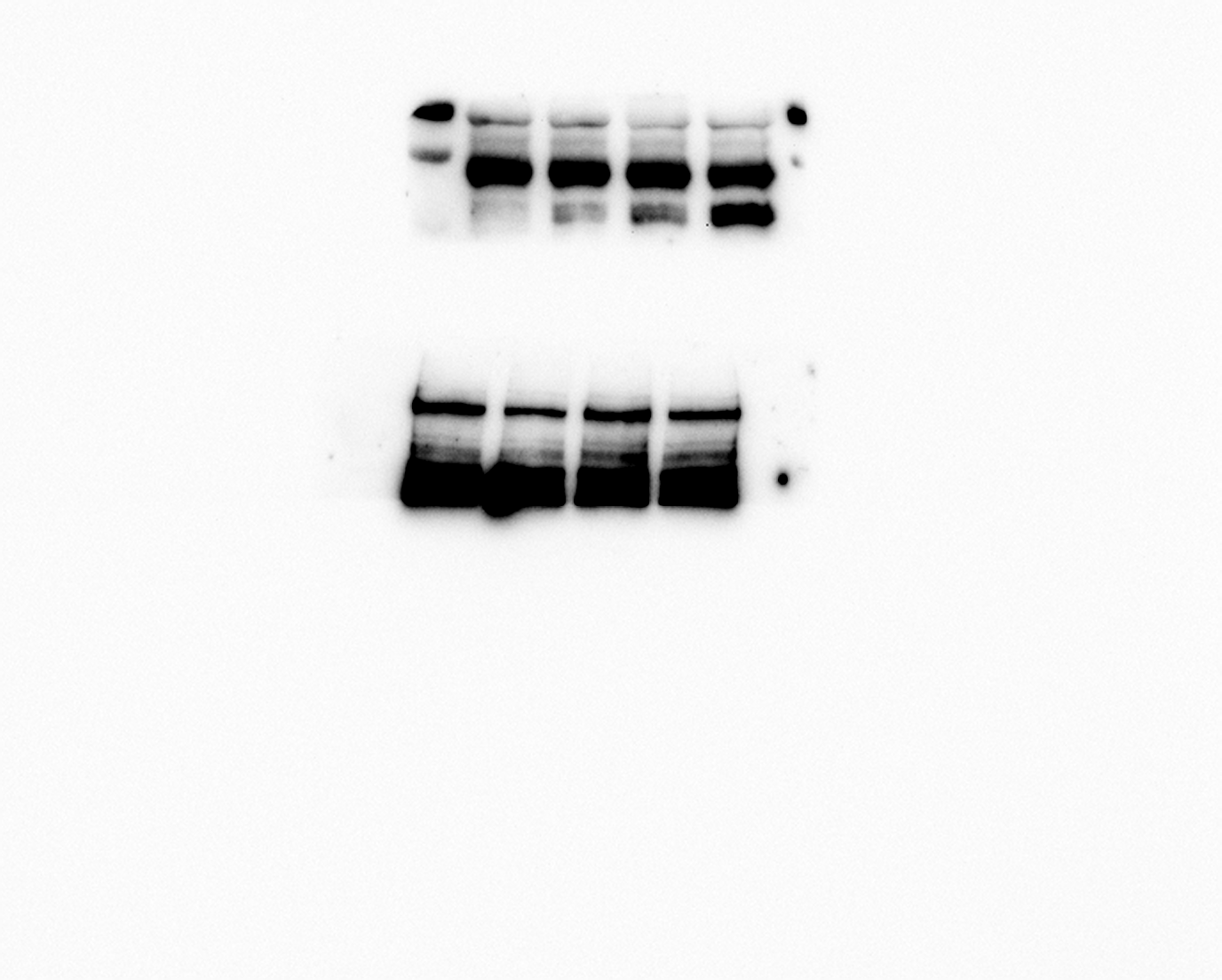

Supplement: Figure 3—source data 2. [file elife-89573-fig3-data2.zip › Figure 3L/ELF3.Tif]

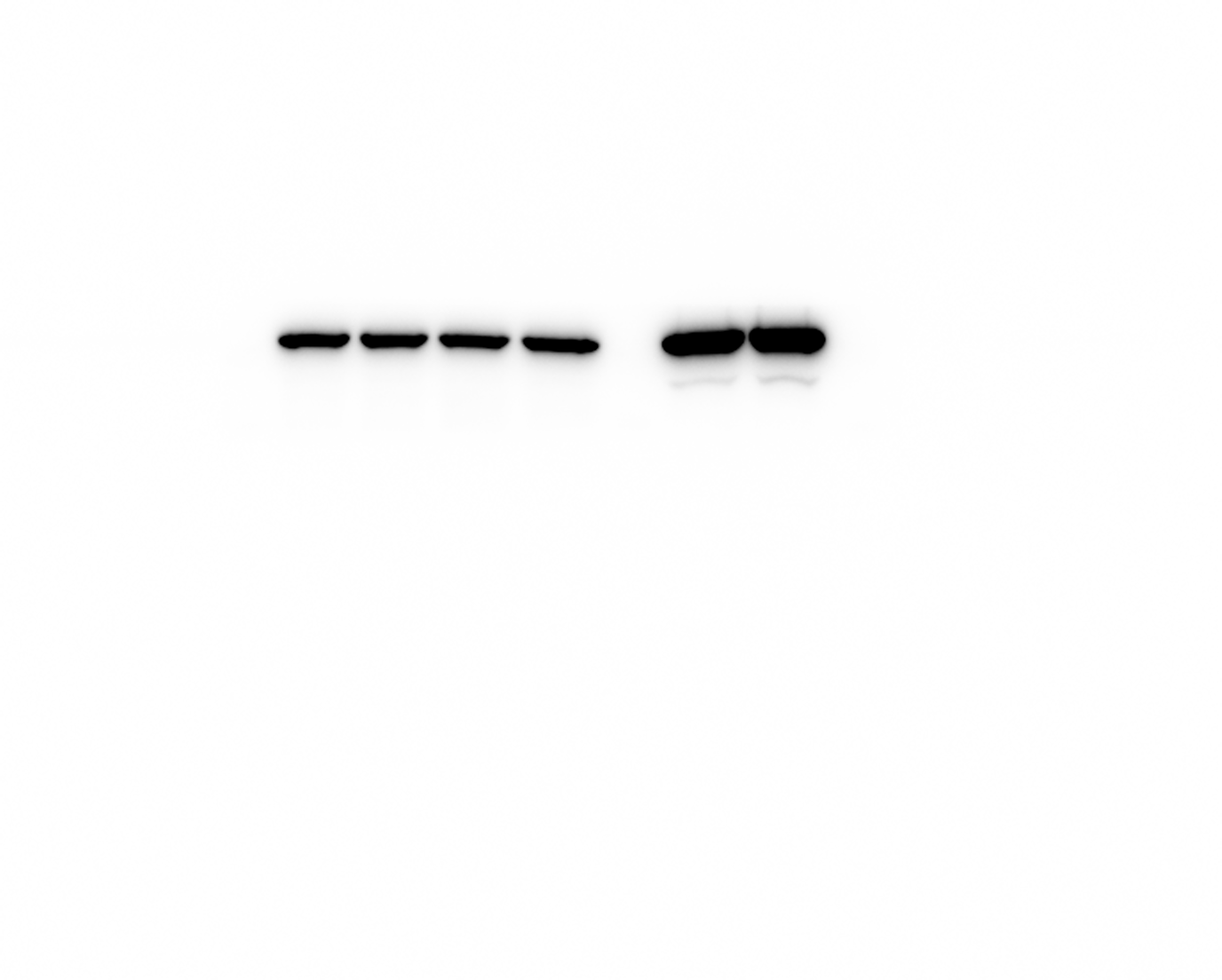

Supplement: Figure 3—source data 2. [file elife-89573-fig3-data2.zip › Figure 3L/GAPDH.Tif]

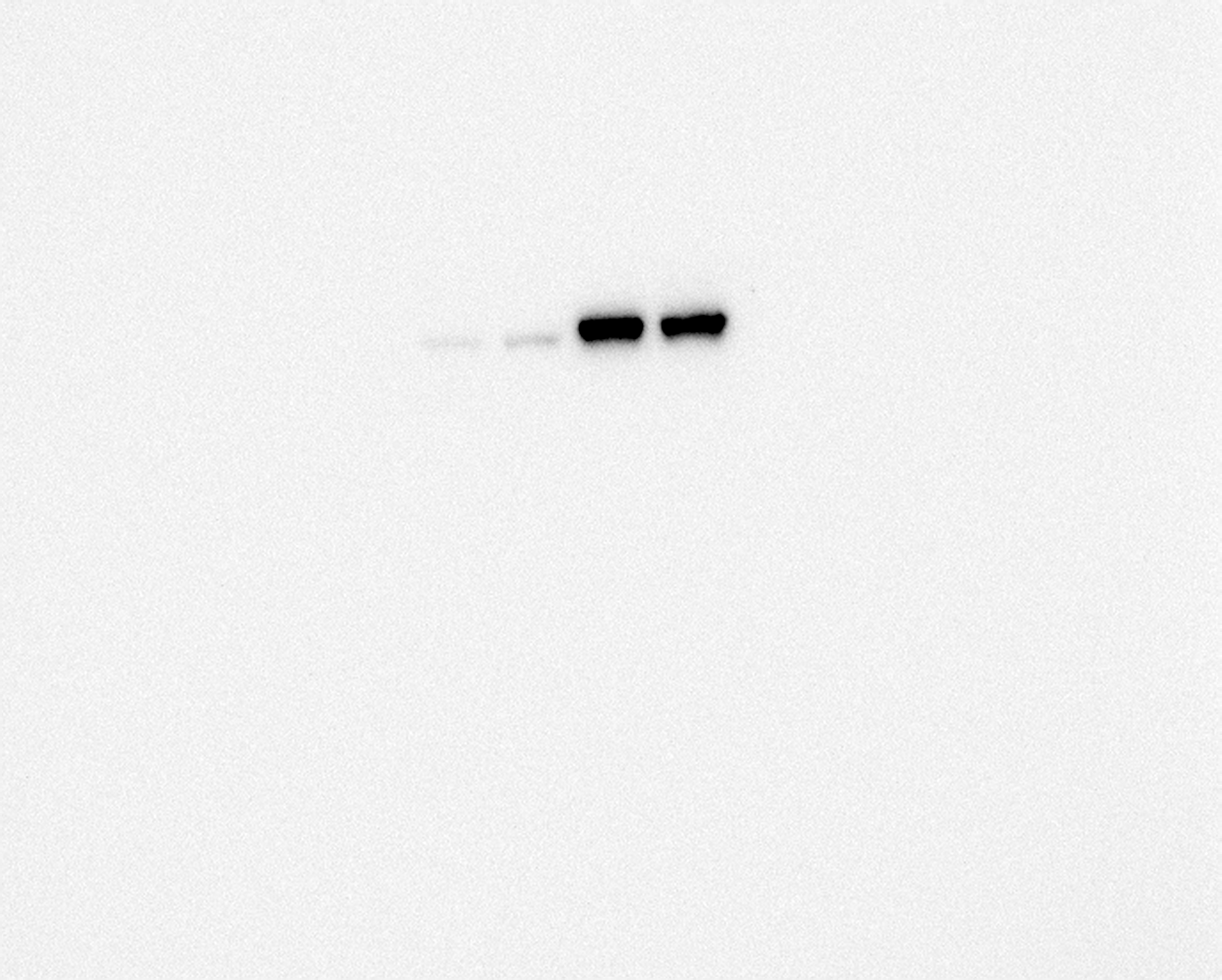

Supplement: Figure 3—source data 2. [file elife-89573-fig3-data2.zip › Figure 3L/p-Chk1.Tif]

Figure 4 Supplement 1A

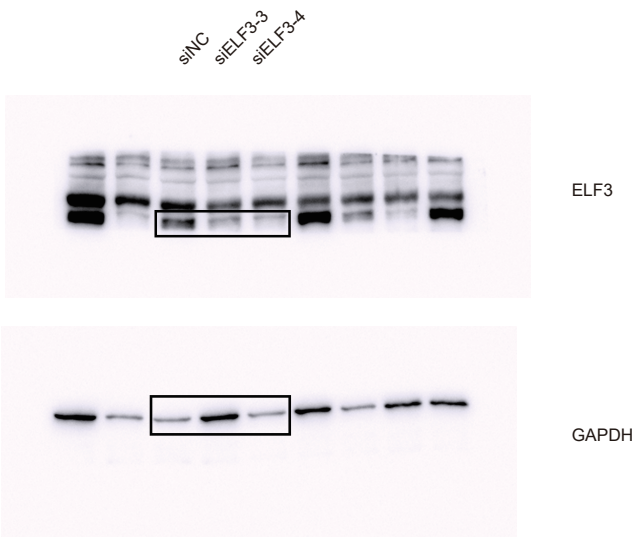

Figure 4 Supplement 1C

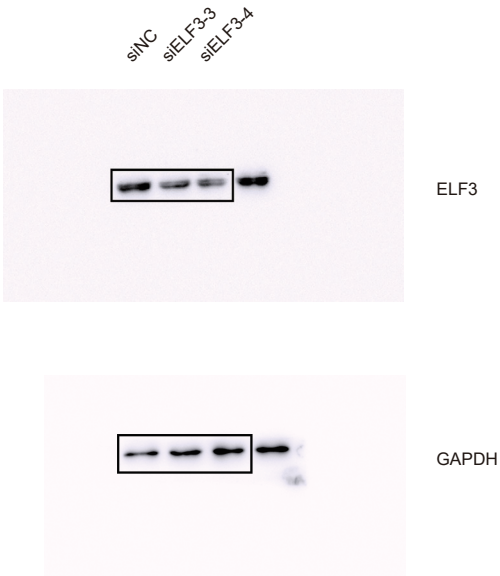

Figure 4 Supplement 1B

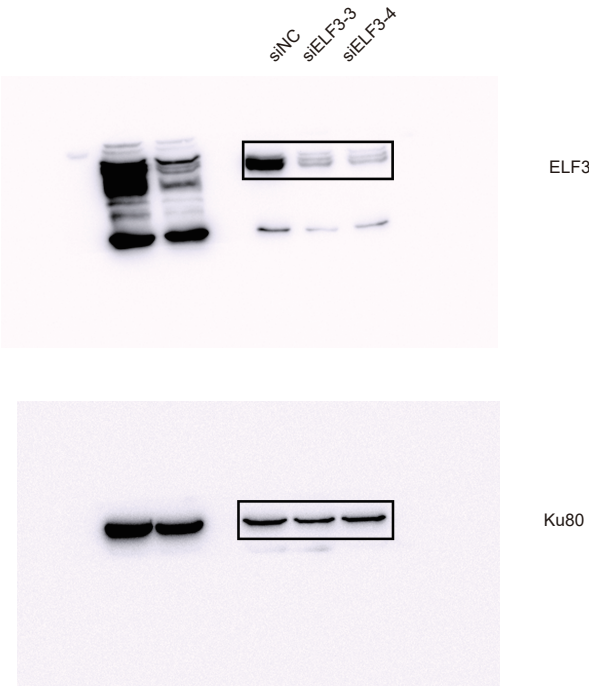

Figure 4 Supplement 1E

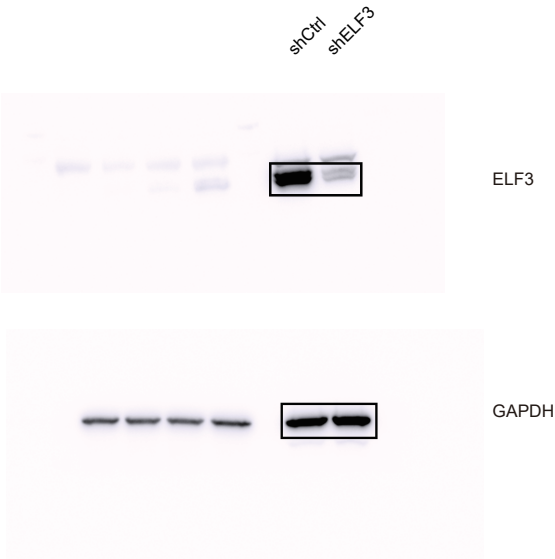

Supplement: Figure 4—figure supplement 1—source data 1. [file elife-89573-fig4-figsupp1-data1.zip › Figure 4 Supplement 1.pdf]

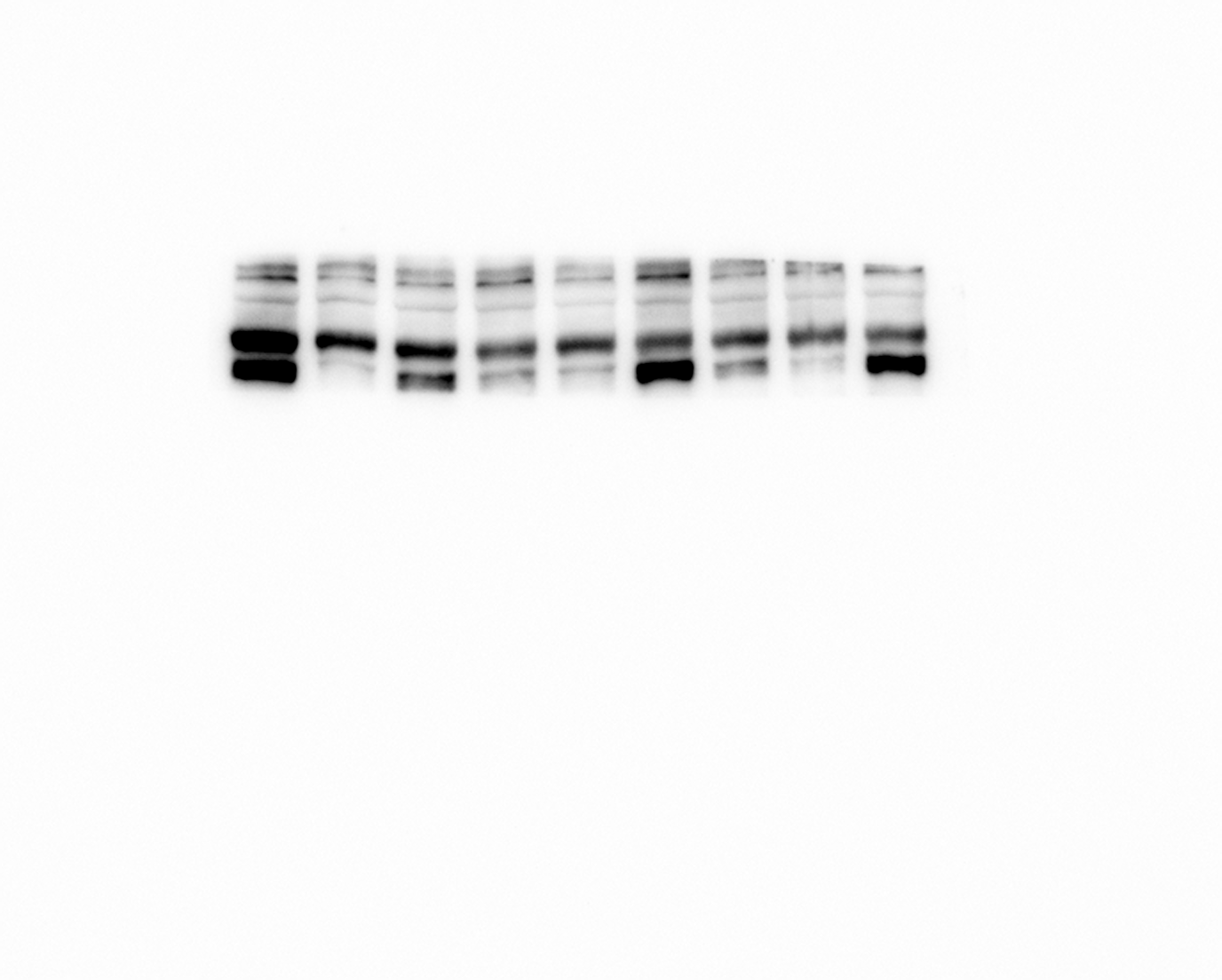

Supplement: Figure 4—figure supplement 1—source data 2. [file elife-89573-fig4-figsupp1-data2.zip › Figure 4 Supplement 1A/ELF3.Tif]

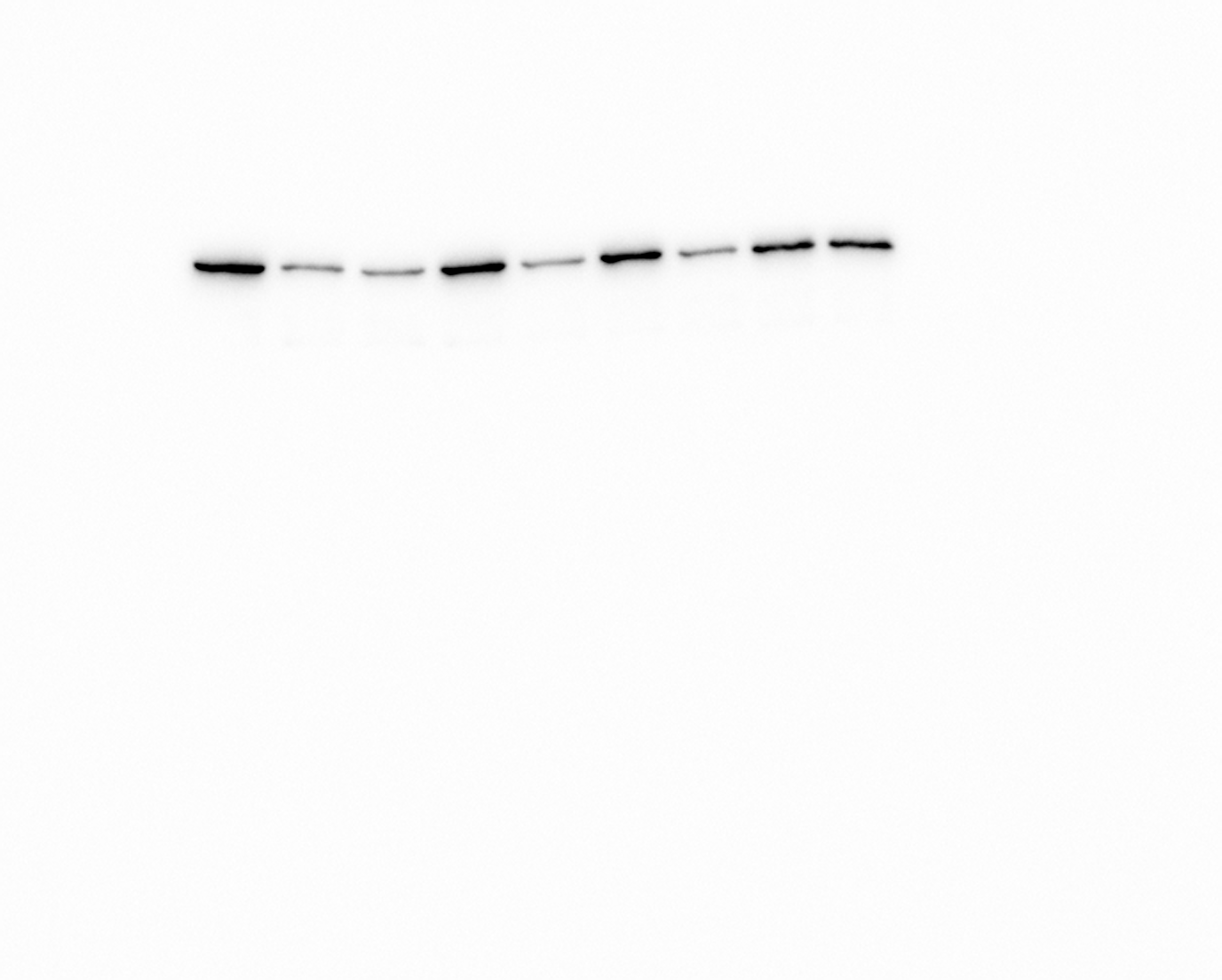

Supplement: Figure 4—figure supplement 1—source data 2. [file elife-89573-fig4-figsupp1-data2.zip › Figure 4 Supplement 1A/GAPDH.Tif]

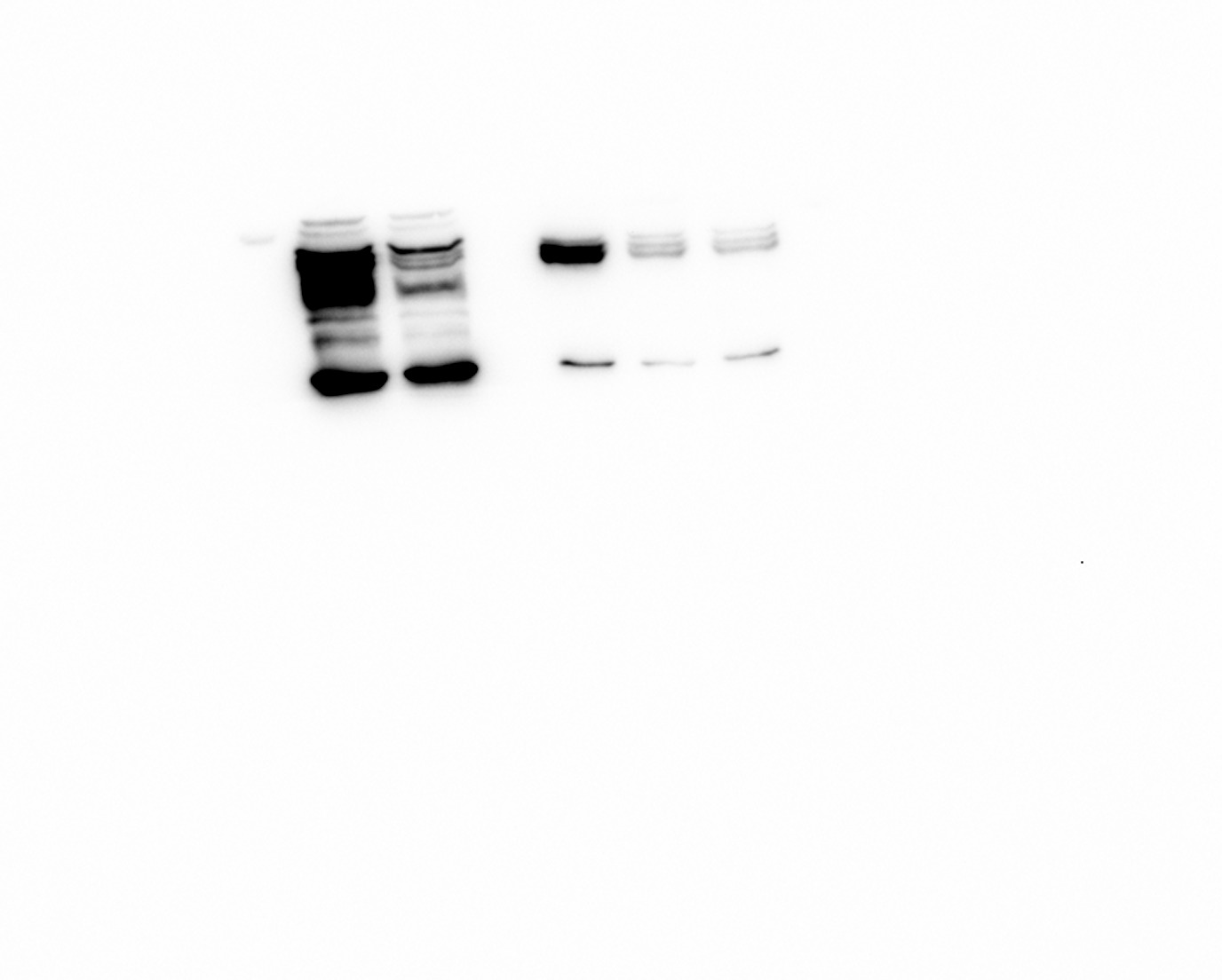

Supplement: Figure 4—figure supplement 1—source data 2. [file elife-89573-fig4-figsupp1-data2.zip › Figure 4 Supplement 1B/ELF3.Tif]

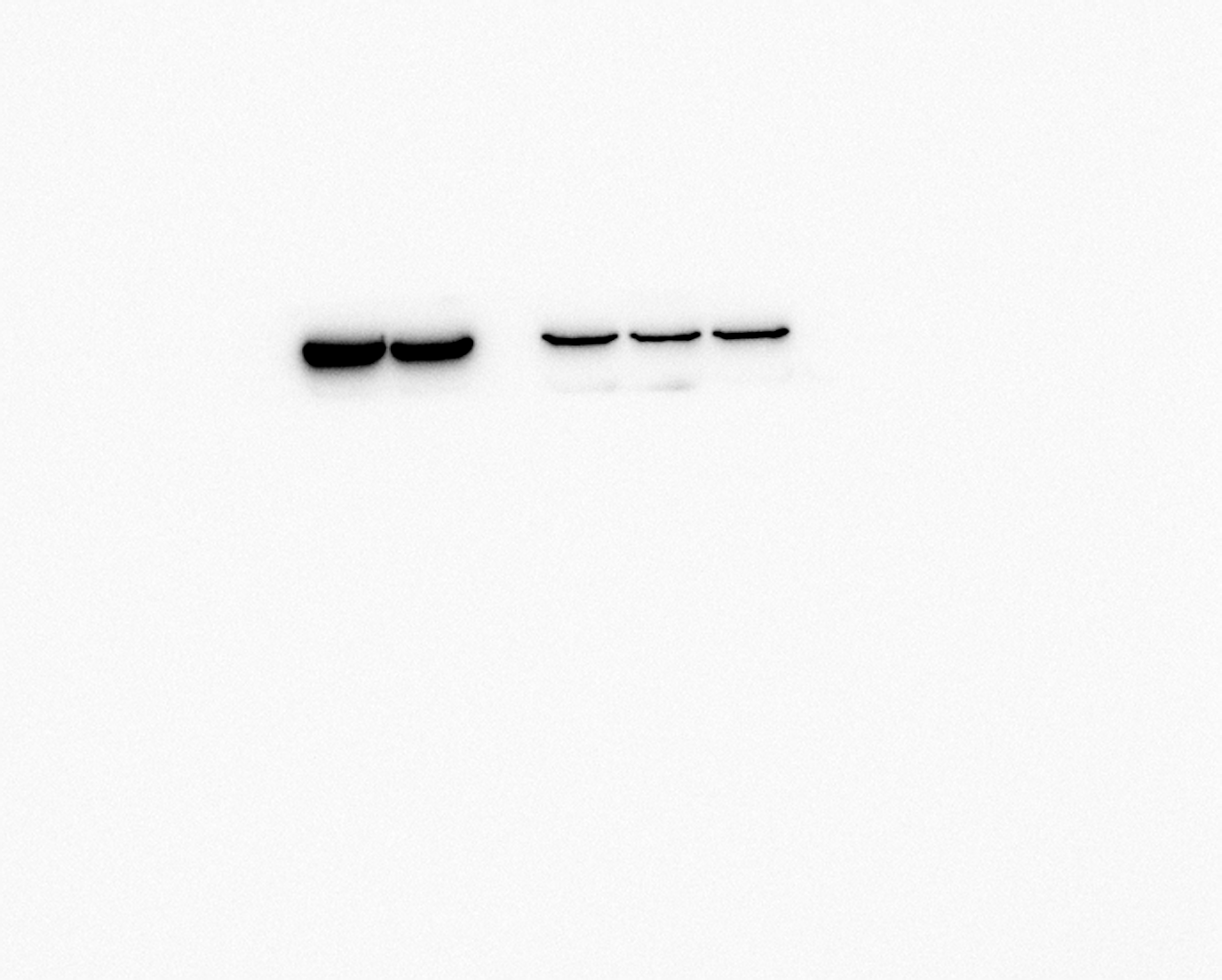

Supplement: Figure 4—figure supplement 1—source data 2. [file elife-89573-fig4-figsupp1-data2.zip › Figure 4 Supplement 1B/Ku80.Tif]

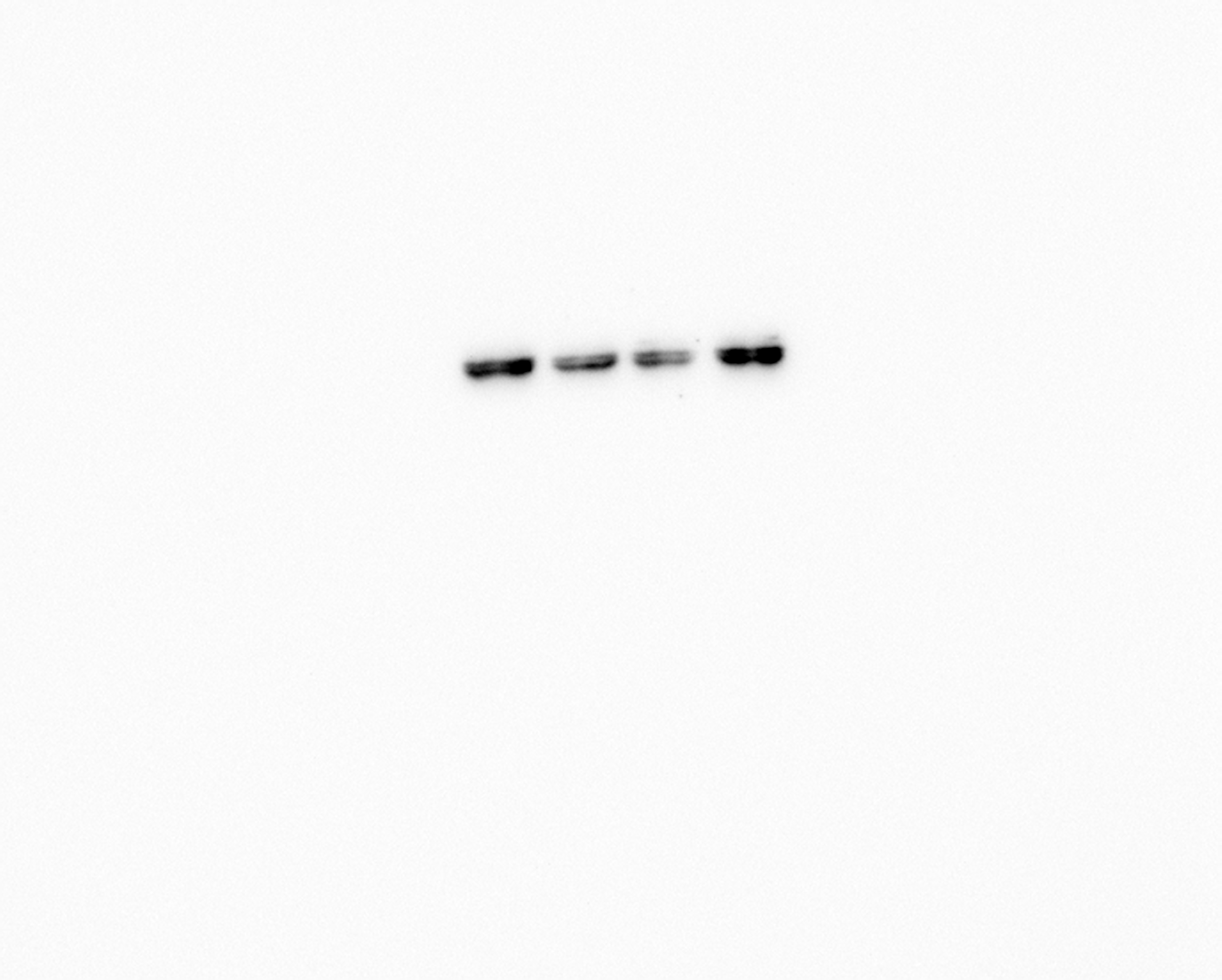

Supplement: Figure 4—figure supplement 1—source data 2. [file elife-89573-fig4-figsupp1-data2.zip › Figure 4 Supplement 1C/ELF3.Tif]

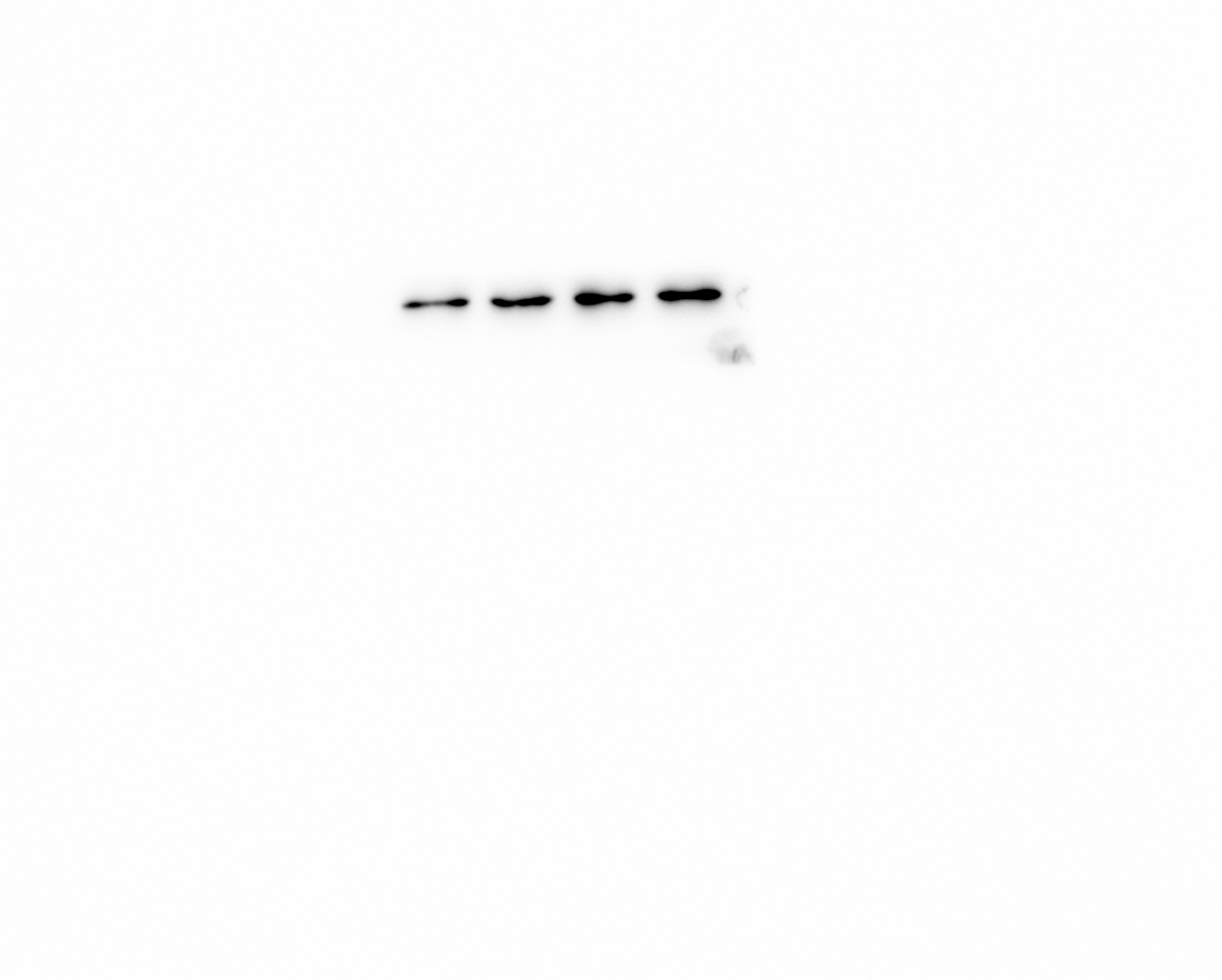

Supplement: Figure 4—figure supplement 1—source data 2. [file elife-89573-fig4-figsupp1-data2.zip › Figure 4 Supplement 1C/GAPDH.Tif]

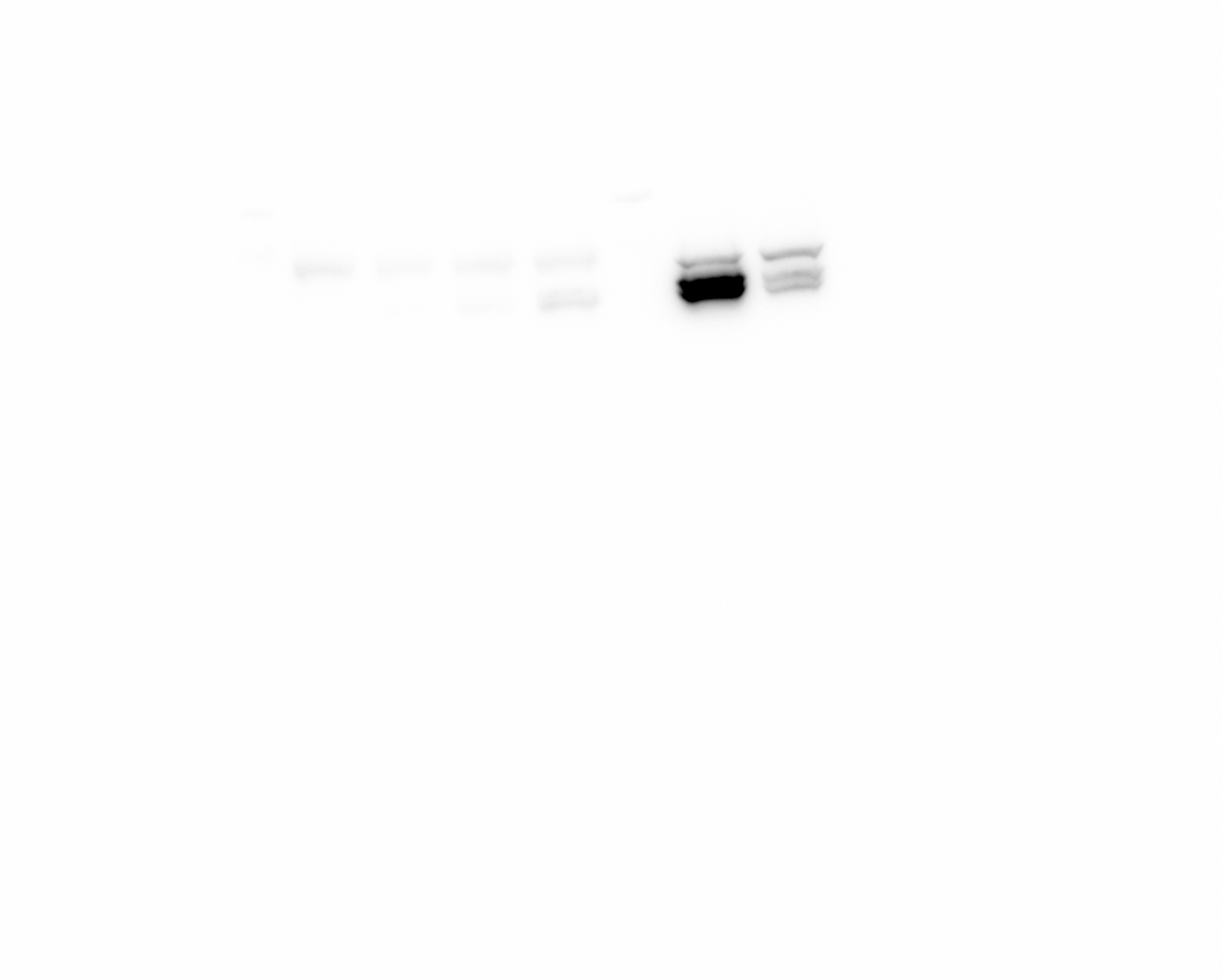

Supplement: Figure 4—figure supplement 1—source data 2. [file elife-89573-fig4-figsupp1-data2.zip › Figure 4 Supplement 1E/ELF3.Tif]

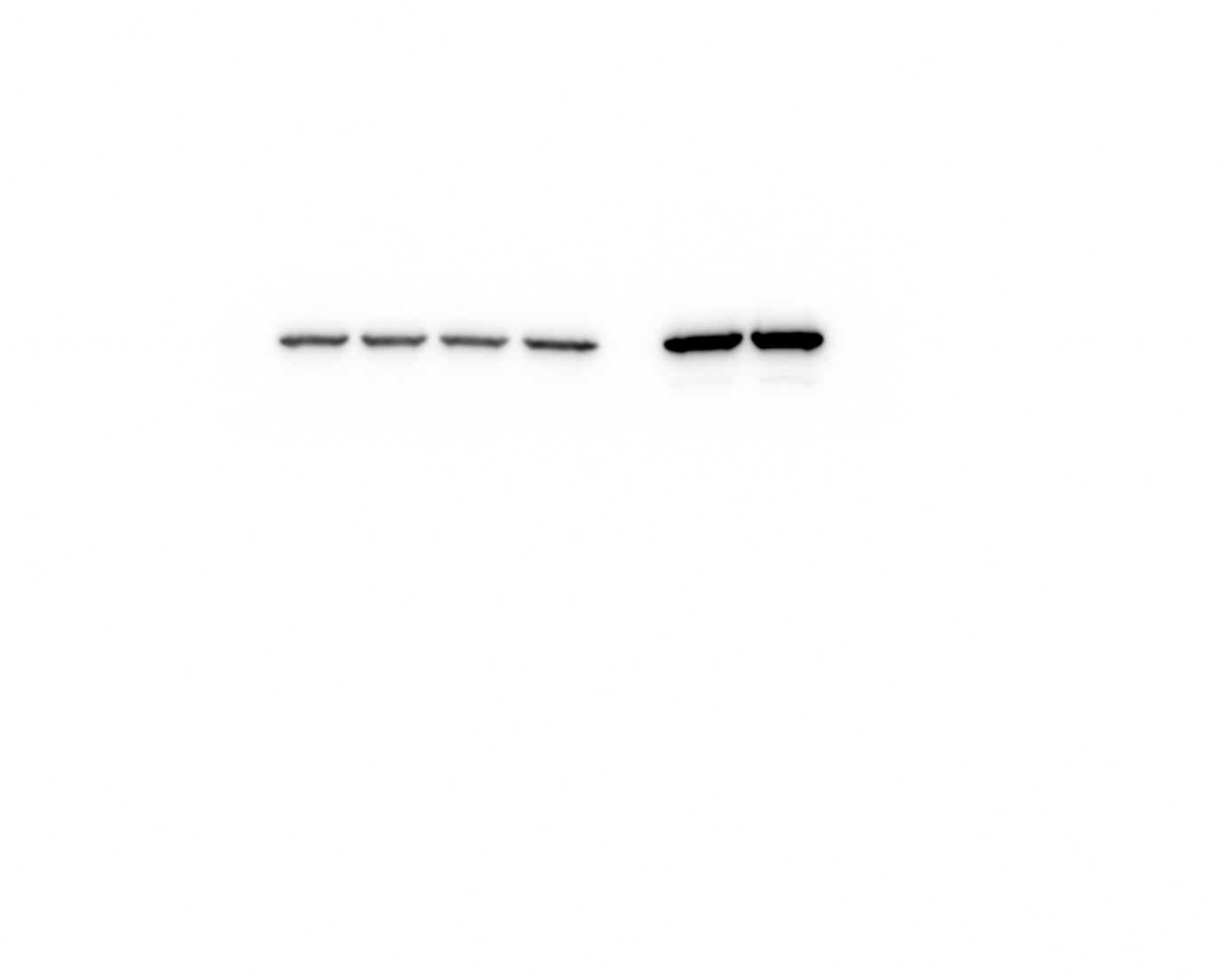

Supplement: Figure 4—figure supplement 1—source data 2. [file elife-89573-fig4-figsupp1-data2.zip › Figure 4 Supplement 1E/GAPDH.Tif]
