# Supplementary material for: Replication stress-inducing ELF3 upregulation promotes BRCA1-deficient breast tumorigenesis in luminal progenitors
Source: eLife. 2026 Jan 7;12:RP89573. doi: 10.7554/eLife.89573 (PMC12779267; doi:10.7554/eLife.89573)
Supplement: Supplementary file 2. [file elife-89573-supp2.docx]

**Supplemental Table S2** **Detailed information on the BRCA1 germline mutation and clinicopathological characteristics of seven patients.**

| **Patient ID** | **BRCA germline mutations** | **age(y)** | **parity** | **pathologic** | **ER** | **PR** | **HER2** | **HER2 FISH** | **Ki-67** | **PAM50 subtype** | **neoadjuvant therapy** |
| --- | --- | --- | --- | --- | --- | --- | --- | --- | --- | --- | --- |
| **Case_1** | c.4013delA (p.Lys1338Argfs*28) | 25 | nulliparous | IDC | 90%+ | negative | 2+ | negative | 20%+ | Luminal A | none |
| **Case_2** | c.2194G>T (p.Glu732Ter) | 43 | multiparous | IMC | 20%+ | negative | 2+ | negative | 70%+ | Luminal B | chemotherapy |
| **Case_3** | c.1069A>T (p.Lys357Ter) | 29 | multiparous | IDC | negative | negative | 2+ | negative | 80%+ | Basal-like | none |
| **Case_4** | c.5251C>T (p.Arg1751Ter) | 43 | multiparous | IDC | negative | negative | 1+ | NA | 60%+ | Basal-like | none |
| **Case_5** | wild-type | 42 | multiparous | IDC | 80%+ | 80%+ | 1+ | NA | 30%+ | Luminal A | none |
| **Case_6** | wild-type | 29 | nulliparous | fibroadenoma | — | — | — | — | — | — | — |
| **Case_7** | wild-type | 33 | nulliparous | fibroadenoma | — | — | — | — | — | — | — |

BRCA1 transcript: NM_007294.3

Abbreviations: ER, estrogen receptor; PR, progesterone receptor; HER2, human epidermal growth factor receptor 2; IDC, invasive ductal carcinoma; IMC, invasive micropapillary carcinoma; FISH, fluorescence in situ hybridization
